# Comparison of a Single-Session Pain Management Skills Intervention With a Single-Session Health Education Intervention and 8 Sessions of Cognitive Behavioral Therapy in Adults With Chronic Low Back Pain: A Randomized Clinical Trial

**DOI:** 10.1001/jamanetworkopen.2021.13401

**Published:** 2021-08-16

**Authors:** Beth D. Darnall, Anuradha Roy, Abby L. Chen, Maisa S. Ziadni, Ryan T. Keane, Dokyoung S. You, Kristen Slater, Heather Poupore-King, Ian Mackey, Ming-Chih Kao, Karon F. Cook, Kate Lorig, Dongxue Zhang, Juliette Hong, Lu Tian, Sean C. Mackey

**Affiliations:** 1 Department of Anesthesiology, Perioperative and Pain Medicine, Stanford University School of Medicine, Palo Alto, California; 2 Feral Scholars, Broaddus, Texas; 3 Department of Rheumatology, Stanford University School of Medicine, Palo Alto, California; 4 Department of Medicine, Northwestern University Feinberg School of Medicine, Chicago, Illinois; 5 Department of Biomedical Data Science, Stanford University School of Medicine, Palo Alto, California

## Abstract

**Importance:**

Chronic low back pain (CLBP), the most prevalent chronic pain condition, imparts substantial disability and discomfort. Cognitive behavioral therapy (CBT) reduces the effect of CLBP, but access is limited.

**Objective:**

To determine whether a single class in evidence-based pain management skills (empowered relief) is noninferior to 8-session CBT and superior to health education at 3 months after treatment for improving pain catastrophizing, pain intensity, pain interference, and other secondary outcomes.

**Design, Setting, and Participants:**

This 3-arm randomized clinical trial collected data from May 24, 2017, to March 3, 2020. Participants included individuals in the community with self-reported CLBP for 6 months or more and an average pain intensity of at least 4 (range, 0-10, with 10 indicating worst pain imaginable). Data were analyzed using intention-to-treat and per-protocol approaches.

**Interventions:**

Participants were randomized to (1) empowered relief, (2) health education (matched to empowered relief for duration and format), or (3) 8-session CBT. Self-reported data were collected at baseline, before treatment, and at posttreatment months 1, 2, and 3.

**Main Outcomes and Measures:**

Group differences in Pain Catastrophizing Scale scores and secondary outcomes at month 3 after treatment. Pain intensity and pain interference were priority secondary outcomes.

**Results:**

A total of 263 participants were included in the analysis (131 women [49.8%], 130 men [49.4%], and 2 other [0.8%]; mean [SD] age, 47.9 [13.8] years) and were randomized into 3 groups: empowered relief (n = 87), CBT (n = 88), and health education (n = 88). Empowered relief was noninferior to CBT for pain catastrophizing scores at 3 months (difference from CBT, 1.39 [97.5% CI, −∞ to 4.24]). Empowered relief and CBT were superior to health education for pain catastrophizing scores (empowered relief difference from health education, −5.90 [95% CI, −8.78 to −3.01; *P* < .001]; CBT difference from health education, −7.29 [95% CI, −10.20 to −4.38; *P* < .001]). Pain catastrophizing score reductions for empowered relief and CBT at 3 months after treatment were clinically meaningful (empowered relief, −9.12 [95% CI, −11.6 to −6.67; *P* < .001]; CBT, −10.94 [95% CI, −13.6 to −8.32; *P* < .001]; health education, −4.60 [95% CI, −7.18 to −2.01; *P* = .001]). Between-group comparisons for pain catastrophizing at months 1 to 3 were adjusted for baseline pain catastrophizing scores and used intention-to-treat analysis. Empowered relief was noninferior to CBT for pain intensity and pain interference (priority secondary outcomes), sleep disturbance, pain bothersomeness, pain behavior, depression, and anxiety. Empowered relief was inferior to CBT for physical function.

**Conclusions and Relevance:**

Among adults with CLBP, a single-session pain management class resulted in clinically significant improvements in pain catastrophizing, pain intensity, pain interference, and other secondary outcomes that were noninferior to 8-session CBT at 3 months.

**Trial Registration:**

ClinicalTrials.gov Identifier: NCT03167086

## Key Points

**Question:**

Is a single-session pain relief class noninferior to 8 sessions of cognitive behavioral therapy (CBT) at 3 months after treatment?

**Findings:**

In this 3-arm randomized clinical trial that included 263 adults with chronic low back pain, a single-session pain management skills class was noninferior to 8 weeks of CBT and superior to a health education class for pain catastrophizing and multiple secondary outcomes at 3 months after treatment.

**Meaning:**

For patients with chronic low back pain, a single-session pain relief skills class showed comparable efficacy to CBT in pain catastrophizing, pain intensity, and pain interference and other outcomes at 3 months after treatment.

## Teaser

This randomized clinical trial compares a single-session pain management skills class with 8 sessions of cognitive behavioral therapy and 1 session of health education for patients with chronic low back pain.

## Introduction

Chronic pain affects an estimated one-third of adults globally.^1^ Chronic low back pain (CLBP) is the most prevalent chronic pain condition^1^ and confers substantial disability, cost, and discomfort. Rates of CLBP are rising despite increased use of medical treatments such as surgery and pharmacology.^2^

A recent expert evidence review concluded that pain education and cognitive behavioral therapy (CBT) should be first-line treatments for CLBP.^3^ Cognitive behavioral therapy for chronic pain engages patients in active pain management^4,5,6^ and typically is delivered by a trained therapist during 8 to 12 group sessions. Cognitive behavioral therapy has small to moderate effects on depressive symptoms,^4^ pain bothersomeness,^5,6^ and pain catastrophizing^5,6,7^—a cognitive and emotional pain response pattern that includes increased attention and feelings of pain helplessness.^8,9^ Pain catastrophizing is associated with CLBP onset^10^ and treatment response,^11^ and decreased pain catastrophizing mediates the effects of CBT.^12,13,14^ Mechanisms of pain catastrophizing include amplified distress and pain-facilitating neural patterns.^15,16,17^ Decreased pain catastrophizing favorably alters brain function and structure^14^ and appears to contribute to improved disability^18,19,20,21^ and activation.^22,23^ Although CBT is effective,^5,6^ multiple barriers can limit patient access, such as time, costs, and therapist availability.^18^ Efficient treatment options are needed, particularly lower-intensity options that may be sufficient for some patients.

We developed a single-session, 2-hour class called *empowered relief* to rapidly equip individuals with pain self-management skills. Empowered relief is rooted in pain-CBT theory and incorporates pain education, self-regulatory skills (ie, relaxation, cognitive reframing, and self-soothing), and mindfulness principles. A noncontrolled pilot study in patients with chronic pain of mixed etiology^19^ (N = 57) revealed moderate to substantial reductions in pain catastrophizing scores at 1 month after treatment with a large effect size (Cohen *d* = 1.15). However, a controlled comparison with CBT remains untested, and durability and scope of the treatment effects are unknown.

Extending this work, we conducted a 3-arm randomized clinical comparative efficacy trial in CLBP to compare (1) empowered relief, (2) a 2-hour health education class, and (3) 8 sessions of pain CBT.^20^ We collected self-report data at baseline, before treatment, and at posttreatment months 1, 2, and 3. We hypothesized that at 3 months after treatment (primary end point), (1) empowered relief would be noninferior to 8-session CBT for pain catastrophizing, (2) empowered relief would be superior to health education for pain catastrophizing, (3) CBT would be superior to health education for pain catastrophizing (assay sensitivity/positive control), and (4) empowered relief would be noninferior to CBT for pain intensity, pain interference, sleep disturbance, pain bothersomeness, pain self-efficacy, physical function, depression, anxiety, and fatigue.

## Methods

### Study Design and Oversight

This clinical trial was performed at a single academic site in the San Francisco Bay Area, California. The trial tested for noninferiority in comparing empowered relief vs CBT and superiority in comparing empowered relief vs health education and CBT vs health education. The study protocol has been published previously (Supplement 1),^20^ was approved by Stanford University’s institutional review board, and followed the Consolidated Standards of Reporting Trials (CONSORT) reporting guideline on noninferiority trials.^21^ Written informed consent was obtained before enrollment. An independent data and safety monitoring committee provided trial oversight. Study data were collected from May 24, 2017, to March 3, 2020.

### Participants

Participants were recruited from the community with advertisements for a no-cost, nondrug study involving 3 treatments for CLBP. A total of $300 compensation was possible for completing the study surveys.

Inclusion criteria consisted of (1) axial low back pain experienced on at least one-half of days in the past 6 months (per the National Institutes of Health Task Force on Research Standards for CLBP^22^), (2) average pain intensity score of at least 4 (range, 0-10, with 10 indicating worst pain imaginable), (3) English fluency, (4) adults aged 18 to 70 years, (5) Pain Catastrophizing Scale score of at least 20 (moderate), and (6) ability to attend as many as eight 2-hour treatment sessions. Exclusion criteria were gross cognitive impairment, radicular symptoms, previous receipt of empowered relief or receipt of CBT in the past 3 years, current substance use disorder, medicolegal factors, suicidal ideation, or severe depression (Mini-International Neuropsychiatric Interview, version 7.0, for screening^23^; the Beck Depression Inventory-II for severity grading^24^; and the Structured Clinical Interview for *DSM-5* Disorders for diagnostics^25^).

### Randomization Procedures and Participant Blinding

Participants were randomly assigned in the Research Electronic Data Capture system^26^ to 1 of 3 groups with no blocking applied. Group allocation was revealed to study staff members and each participant after enrollment.

### Data Protections and Investigator Blinding

Participant identification was protected with a unique study identification number. All data were received electronically, instantly locked in the database, and stored with double-password protection. The project manager (A.R.) and treatment instructors (M.S.Z., K.S., and H.P.-K.) were unblinded to individual group assignment; coinvestigators (B.D.D., D.S.Y., I.M., M.-C.K., K.F.C., K.L., D.Z., J.H., L.T., and S.C.M.) were blinded until the 3-month data were received.

### Assessment Times

All measures were administered after study enrollment. To minimize regression to the mean effects, a second full assessment (minus demographics; administered 3 days before treatment) was the pretreatment baseline. Posttreatment assessments (months 1, 2, and 3) mirrored the pretreatment baseline survey (minus treatment expectations).

### Outcomes

The primary efficacy outcome for noninferiority and superiority analyses was Pain Catastrophizing Scale score at 3 months after treatment. The 13-item Pain Catastrophizing Scale^27^ measures the frequency of various cognitive or emotional responses to pain (eg, “It’s awful and I feel that it overwhelms me”). Responses range from 0 (not at all) to 4 (all the time); sum scores range from 0 to 52. The scale has good psychometric consistency^28,29,30,31,32^ and a high coefficient α (0.87).^27^

Secondary outcomes included (1) mean pain intensity during the previous 7 days^33^; (2) National Institutes of Health Patient-Reported Outcomes Measurement Information System short-form measures to evaluate pain interference, sleep disturbance, pain behavior, depression, anxiety, physical function, and fatigue^20^; (3) pain bothersomeness during the previous 7 days (range, 0 [not at all bothersome] to 10 [extremely bothersome])^5,6^; (4) pain self-efficacy via the Pain Self-Efficacy Questionnaire (range, 0 [not at all confident] to 60 [completely confident])^34^; and (5) treatment expectations, assessed after group assignment, using the Stanford Expectations of Treatment Scale^35^ at 3 months after treatment. Table 1 shows additional clinical characteristics and treatment history data.

**Table 1.** Baseline Demographic and Clinical Characteristics by Treatment Group

Characteristic	Patient group^a^			
	Total sample (N = 263)	Empowered relief (n = 87)	CBT (n = 88)	Health education (n = 88)
Age, mean (SD), y	47.9 (13.8)	49.7 (15.0)	45.9 (13.1)	48.0 (13.2)
Sex				
Female	131 (49.8)	44 (50.6)	40 (45.5)	47 (53.4)
Male	130 (49.4)	42 (48.3)	47 (53.4)	41 (46.6)
Other	2 (0.8)	1 (1.1)	1 (1.1)	0
Race				
White	157 (60.2)	57 (66.3)	48 (54.5)	52 (59.8)
Asian/Pacific Islander	64 (24.5)	16 (18.6)	27 (30.7)	21 (24.1)
African American	11 (4.2)	5 (5.8)	4 (4.5)	2 (2.3)
American Indian/Alaska Native	2 (0.8)	1 (1.2)	0	1 (1.1)
Other^b^	27 (10.3)	7 (8.1)	9 (10.2)	11 (12.6)
Ethnicity				
Hispanic	17 (6.6)	7 (8.3)	6 (6.9)	4 (4.6)
Non-Hispanic	241 (93.4)	77 (91.7)	81 (93.1)	83 (95.4)
Relationship status				
Married/cohabitating	160 (60.8)	53 (60.9)	58 (65.9)	49 (55.7)
Never married	71 (27.0)	27 (31.0)	22 (25.0)	22 (25.0)
Divorced	22 (8.4)	5 (5.7)	4 (4.5)	13 (14.8)
Separated	6 (2.3)	1 (1.1)	1 (1.1)	4 (4.5)
Widowed	4 (1.5)	1 (1.1)	3 (3.4)	0
Educational attainment				
High school	6 (2.3)	2 (2.3)	2 (2.3)	2 (2.3)
Some college	66 (25.1)	23 (26.4)	17 (19.3)	26 (29.5)
Bachelor’s degree	90 (34.2)	30 (34.5)	32 (36.4)	28 (31.8)
Master’s degree	67 (25.5)	21 (24.1)	26 (29.5)	20 (22.7)
Doctoral degree	34 (12.9)	11 (12.6)	11 (12.5)	12 (13.6)
Employment				
Full-time	114 (46.2)	30 (36.1)	45 (53.6)	39 (48.8)
Part-time	49 (19.8)	17 (20.5)	14 (16.7)	18 (22.5)
Retired	39 (15.8)	22 (26.5)	9 (10.7)	8 (10.0)
Student	16 (6.5)	6 (7.2)	6 (7.1)	4 (5.0)
Unemployed	16 (6.5)	4 (4.8)	6 (7.1)	6 (7.5)
Disabled	13 (5.3)	4 (4.8)	4 (4.8)	5 (6.3)
Household income, $				
<30 000	31 (12.3)	14 (16.5)	7 (8.5)	10 (11.6)
<50 000	27 (10.7)	9 (10.6)	7 (8.5)	11 (12.8)
<70 000	30 (11.9)	12 (14.1)	10 (12.2)	8 (9.3)
≥70 000	165 (65.2)	50 (58.8)	58 (70.7)	57 (66.3)
Smoking status				
Never smoked	175 (66.8)	58 (66.7)	65 (73.9)	52 (59.8)
Current	20 (7.6)	5 (5.7)	6 (6.8)	9 (10.3)
Past	67 (25.6)	24 (27.6)	17 (19.3)	26 (29.9)
BMI, mean (SD)	27.0 (6.3)	27.3 (6.0)	27.0 (6.5)	26.7 (6.3)
Pain duration				
6-12 mo	14 (5.3)	4 (4.6)	8 (9.1)	2 (2.3)
1-5 y	79 (30.0)	25 (28.7)	24 (27.3)	30 (34.1)
>5 y	170 (64.6)	58 (66.7)	56 (63.6)	56 (63.6)
Back pain intensity score in past 30 d, mean (SD)^c^	5.8 (1.3)	5.6 (1.3)	5.9 (1.3)	6.0 (1.3)
Treatment expectations, mean (SD)^d^				
Positive	3.69 (1.27)	3.71 (1.30)	3.74 (1.22)	3.60 (1.32)
Negative	2.14 (1.30)	2.29 (1.34)	2.00 (1.12)	2.12 (1.43)
Comorbid pain condition^e^				
1	127 (48.3)	38 (43.7)	43 (48.9)	46 (52.3)
≥2	48 (18.3)	18 (20.7)	15 (17.0)	15 (17.0)
Fibromyalgia	10 (3.8)	2 (2.3)	4 (4.5)	4 (4.5)
Complex regional pain syndrome	3 (1.1)	1 (1.1)	0	2 (2.3)
Pelvic pain	22 (8.4)	4 (4.6)	9 (10.2)	9 (10.2)
Migraine	31 (11.8)	13 (14.9)	8 (9.1)	10 (11.4)
Other	160 (60.8)	54 (62.1)	54 (61.4)	52 (59.1)
Medication use for CLBP				
Opioids	43 (16.3)	13 (14.9)	17 (19.3)	13 (14.8)
NSAID/acetaminophen^f^	123 (46.8)	45 (51.7)	38 (43.2)	40 (45.5)
Adjunctive pain medications^g^	66 (25.1)	22 (25.3)	29 (33.0)	15 (17.0)
Mental health disorders				
Mood disorders				
Ever	132 (50.2)	44 (50.6)	44 (50.0)	44 (50.0)
Current	16 (6.1)	9 (10.3)	4 (4.5)	3 (3.4)
Past	131 (49.8)	43 (49.4)	44 (50.0)	44 (50.0)
Anxiety disorders	82 (31.2)	24 (27.6)	33 (37.5)	25 (28.4)

Abbreviations: BMI, body mass index (calculated as weight in kilograms divided by square of height in meters); CBT, cognitive behavioral therapy; CLBP, chronic low back pain; NSAID, nonsteroidal anti-inflammatory drug.

^a^ Unless otherwise indicated, data are expressed as No. (%) of patients. Numbers may not total those in column headings owing to missing data. Analysis uses the intention-to-treat approach. Wald χ^2^ test was used to compare categorical variables; F test, for continuous variables. All differences between groups were nonsignificant.

^b^ Racial/ethnic categories were not specified.

^c^ Scores range from 0 to 10, with 10 indicating worst pain imaginable.

^d^ Scored using the Stanford Expectations of Treatment Scale; scores range from 1 to 7, with higher scores indicating greater expectations.

^e^ Indicates pain conditions comorbid with chronic low back pain, excluding postsurgical pain.

^f^ Prescription and/or over-the-counter (OTC).

^g^ Neuropathic pain medication, muscle relaxant, and all other pain-related medication (OTC or prescription).

### Study Group Interventions

#### Empowered Relief

Empowered relief consists of a single-session, 2-hour pain class that includes pain neuroscience education, mindfulness principles, and CBT skills (identifying distressing thoughts and emotions, cognitive reframing, a relaxation response exercise, and a self-soothing action plan).^19,20^ The manualized class was delivered by trained instructors (with doctoral psychology degrees [M.S.Z. and K.S.]) to cohorts using an electronic slide deck. Participants also received a 20-minute relaxation MP3 audio file with binaural tones. Treatment fidelity checklists were completed at the class.^20^

#### Health Education

The health education class was matched to empowered relief on 4 key factors: duration, structure, format, and site.^36^ Health education was delivered by a single instructor with a master’s degree in public health and skill in treatment research using an electronic slide deck. Content included warning signs of back pain, when to speak with a physician, general nutrition, and medication managment.^36^ Treatment fidelity checklists were completed at the class.^20^

#### Cognitive Behavioral Therapy

The CBT group attended eight 2-hour sessions delivered by doctoral psychologists (including H.P.-K.). The specific CBT protocol has been tested in large CLBP clinical trials.^5,6,7,37^ Content of CBT is detailed elsewhere^7^ and spans a range of topics and pain relief skills. Participants received a workbook, 2 relaxation audio files, and an optional book.^38^ Attendance at 5 sessions determined completer status. Treatment fidelity checklists were completed at every class.^20^

### Sample Size Calculation

Calculations indicated that 231 participants would ensure 165 completers at 3 months after treatment. To test the primary hypothesis of noninferiority of empowered relief vs CBT, 55 participants per arm provides 80% power with a noninferiority margin of 4.3 (preselected because it is 50% of the Pain Catastrophizing Scale difference between CBT and health education^39^ at a 1-sided significance level of .025, assuming no difference between empowered relief and CBT). To test our superiority hypothesis and compare the mean difference in pain catastrophizing score at month 3 after treatment between the empowered relief and health education groups and between the CBT and health education groups, 55 participants per arm provides 80% power for detecting a mean difference of 0.595 SD, which is 4.74 assuming an SD of 8.0 based on a 2-sample *t* test at a significance level of .025. The 2-sided significance level of .025 accounts for 2 comparisons. The longitudinal analysis using more information is expected to provide more power than the 2-sample *t* test.

### Statistical Analysis

Continuous and categorical baseline variables were summarized using mean (SD) and count (proportions), respectively. The standardized mean differences between treatment groups were calculated. Between-group comparisons were conducted using the analysis of variance or Fisher exact test, when appropriate. Intention-to-treat analysis was used to investigate the causal effect of the treatment. For evaluating the treatment effect of empowered relief and CBT on pain catastrophizing, mixed models for repeated measure (MMRM) regression analysis was conducted with the dependent variable being pain catastrophizing scores at baseline and months 1 to 3 and independent variables being time (baseline, months 1-3 [categorical variables]) and interactions between posttreatment months 1 to 3 and treatment groups (empowered relief, CBT, and health education [categorical variables]). The unstructured covariance among pain catastrophizing scores was used. Treatment effects were summarized as the estimated between-group difference in pain catastrophizing scores at posttreatment month 3 and the associated 95% CI. Because the MMRM analysis included baseline pain catastrophizing scores in repeated measurements, the estimated treatment effect was adjusted for potential imbalance in pain catastrophizing score at baseline. Noninferiority of empowered relief to CBT was evaluated based on the 1-sided 97.5% CI for between-group differences with a noninferiority margin of 4.3 points. The estimated treatment effect at posttreatment months 1 and 2 based on MMRM analysis was also summarized. Similar analyses examined the treatment effect for all secondary outcomes at each posttreatment month. As sensitivity analysis, per protocol analysis was used as a more conservative test of noninferiority of empowered relief to CBT. The additional intention-to-treat MMRM analysis adjusting for age, sex, race, body mass index, duration of back pain, educational attainment, mental health diagnosis, and comorbid pain conditions was also conducted as part of the sensitivity analysis. For the primary end point, the statistical significance level for comparing CBT with health education and empowered relief with health education was set at a 2-sided level of .025 to adjust for 2 comparisons. The noninferiority test was based on the 1-sided 97.5% CI. For the priority secondary end points (pain intensity and interference), the α level for the superiority test (CBT vs health education and empowered relief vs health education) was set at .0125 to adjust for 4 comparisons. The noninferiority was based on 1-sided 98.75% CI to adjust for 2 end points. All other secondary end points were set at a 2-sided level of .05. To assess the missing-at-random assumptions required for the MMRM analysis, we summarized the attrition rate by treatment group at each stage and compared baseline characteristics between those who completed posttreatment surveys and those who did not. Analyses were performed with SAS Enterprise Guide, version 7.15 (SAS Institute, Inc).

## Results

### Participants

The enrolled sample in this randomized clinical trial included 263 individuals (131 women [49.8%], 130 men [49.4%], and 2 other [0.8%]; mean [SD] age, 47.9 [13.8] years) who were predominantly White (157 of 261 [60.2%]), non-Hispanic (241 of 258 [93.4%]), married or cohabitating (160 of 263 [60.8%]), with at least some college education (257 of 263 [97.7%]), with CLBP duration of more than 5 years (170 of 263 [64.6%]), and with at least 1 comorbid chronic pain condition (127 of 263 [48.3%]). Participants were randomized into 3 groups: empowered relief (n = 87), CBT (n = 88), and health education (n = 88). The Stanford Expectations of Treatment Scale revealed overall low mean (SD) negative (2.29 [1.34]) and positive (3.71 [1.30]) treatment expectations with no group differences. Table 1 displays the baseline characteristics by treatment group.

**Figure 1.**
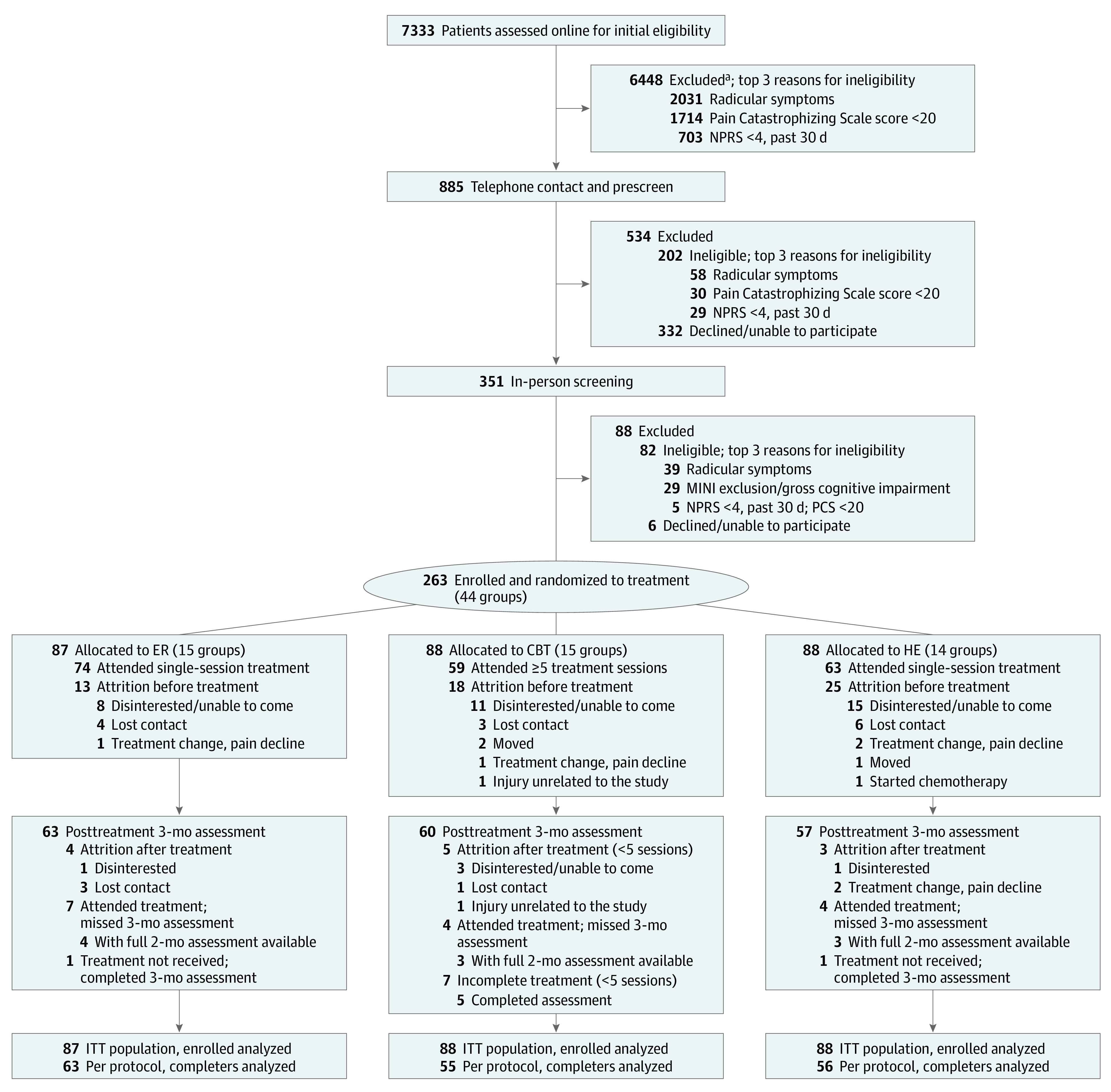
CONSORT Participant Flow CBT indicates cognitive behavioral therapy; CONSORT, Consolidated Standards of Reporting Trials; ER, empowered relief; HE, health education; ITT, intention to treat; MINI, Mini-International Neuropsychiatric Interview; NPRS, Numeric Pain Rating Scale. ^a^Based on online eligibility screen.

Figure 1 presents the CONSORT diagram and participant flow. A total of 7333 individuals completed online eligibility screening, and 885 met the basic eligibility criteria. Of those, 534 were ineligible after a telephone screen. The remaining 351 visited the study site for a final eligibility assessment involving physical examination (for radicular symptoms) and a stepwise depression assessment. Those with severe depression were excluded and offered local resources. The pretreatment attrition rate by group was 28.4% (25 of 88) for health education, 20.5% (18 of 88) for CBT, and 14.9% (13 of 87) for empowered relief.

### Efficacy

**Figure 2.**
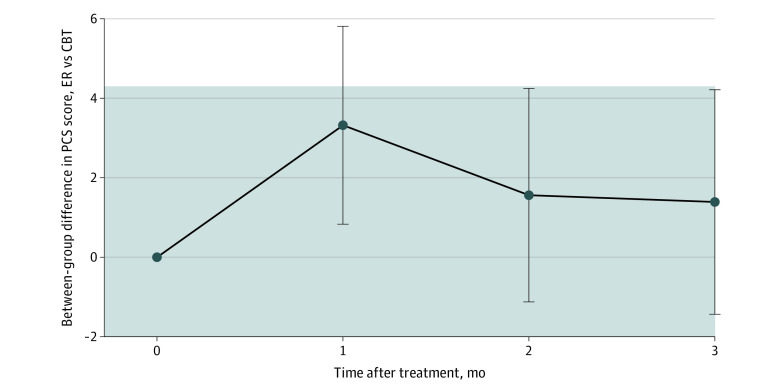
Difference in Pain Catastrophizing Scale (PCS) Score Over Time CBT indicates cognitive behavioral therapy; ER, empowered relief. Gray band displays the noninferiority margin.

Using intention-to-treat analysis, we found noninferiority for 2-hour empowered relief compared with 16-hour CBT for pain catastrophizing scores achieved at 3 months after treatment. Table 2 displays the pain catastrophizing score between-group difference of 1.39 (97.5% CI, −∞ to 4.24), which did not exceed the prespecified noninferiority margin of 4.3, a notably more stringent margin than the minimally important difference of 6.8 cited in the literature^40^ (Figure 2).

**Table 2.** Between-Group Differences in Posttreatment Pain Catastrophizing Scale Scores^a^

Time	Mean (SD) score [No. of patients]^b^			CBT vs heath education		Empowered relief vs health education		Empowered relief vs CBT	
	Empowered relief	CBT	Health education	Estimate (SE) [95% CI]	*P* value^c^	Estimate (SE) [95% CI]	*P* value^c^	Estimate (SE) [one-sided 97.5% CI]	*P* value^c^
Baseline	22.09 (9.84) [78]	23.01 (8.98) [76]	24.81 (10.32) [69]	NA	NA	NA	NA	NA	NA
Posttreatment									
Month 1	15.49 (8.90) [65]	12.53 (8.26) [59]	19.98 (9.60) [57]	−6.93 (1.31) [−9.51 to −4.35]	<.001	−3.62 (1.28) [−6.14 to −1.09]	.0052	3.32 (1.27) [−∞ to 5.81]	.009
Month 2	13.95 (9.78) [63]	12.57 (8.09) [60]	19.36 (10.32) [56]	−6.08 (1.42) [−8.87 to −3.28]	<.001	−4.52 (1.40) [−7.28 to −1.76]	.002	1.56 (1.37) [−∞ to 4.26]	.26
Month 3	13.17 (10.15) [64]	11.87 (9.25) [61]	19.74 (9.95) [58]	−7.29 (1.48) [−10.20 to −4.38]	<.001	−5.90 (1.46) [−8.78 to −3.01]	<.001	1.39 (1.44) [−∞ to 4.24]	.34

Abbreviations: CBT, cognitive behavioral therapy; NA, not applicable.

^a^ Scores range from 0 to 52, with higher scores indicating more frequent cognitive or emotional responses to pain. Negative values indicate decreased Pain Catastrophizing Scale score. Analysis uses the intention-to-treat approach.

^b^ Indicates the number of participants with observed outcome (Pain Catastrophizing Scale score).

^c^ Calculated using 2-sided Wald test.

Clinically meaningful pain catastrophizing scores were found for empowered relief and CBT (empowered relief, −9.12 [95% CI, −11.6 to −6.67; *P* <.001]; CBT, −10.94 [95% CI, −13.6 to −8.32; *P* < .001]; health education, −4.60 [95% CI, −7.18 to −2.01; *P* < .001]). Table 2 displays all between-group comparisons for pain catastrophizing at months 1 to 3 adjusted for baseline pain catastrophizing scores and using intention-to-treat analysis. Empowered relief was superior to health education (difference in pain catastrophizing score at month 3, −5.90 [95% CI, −8.78 to −3.01; *P* < .001]). Cognitive behavioral therapy was superior to health education, with a difference in catastrophizing score of −7.29 at month 3 (95% CI, −10.20 to −4.38; *P* < .001). The statistical significance remained after Bonferroni adjustment of 2 comparisons, because both *P* values are less than .05/2 = .025 (8.41 × 10^−7^ for CBT vs health education and 5.32 × 10^−5^ for empowered relief vs health education). eTable 1 in Supplement 2 displays the per protocol comparisons, and the difference between empowered relief and CBT is 1.54 (97.5% CI, −∞ to 4.29) at posttreatment month 3, confirming the noninferiority of empowered relief.

eTable 2 in Supplement 2 provides the selected minimally important difference margins and justifications for all secondary outcomes. Table 3 reports the between-group posttreatment comparisons for all secondary outcomes. At 3 months, empowered relief evidenced noninferiority to CBT for pain intensity and pain interference (our prioritized secondary outcomes) with correction applied for multiple comparisons (1-sided 98.75% CI, −∞ to 0.63 for pain intensity and 1-sided 98.75% CI, −∞ to 3.53 for pain interference). Empowered relief was noninferior to CBT for sleep disturbance, depression, anxiety, pain behavior, and pain bothersomeness but not for self-efficacy and fatigue. Empowered relief was inferior to CBT for physical function. Both empowered relief and CBT were superior to health education at 3 months after treatment on all secondary outcomes except for fatigue for empowered relief vs health education. Given that the nonpriority secondary outcome evaluations were considered exploratory, no correction was made for type I errors. eTable 3 in Supplement 2 reports between-group comparisons for all secondary outcomes with adjustment for baseline covariates and outcome variables; the results remain consistent with those in Table 3, although some associations become less statistically significant, potentially owing to lack of power.

**Table 3.** Secondary Outcomes at Baseline and Posttreatment Months 1 to 3 by Treatment Group With Between-Group Comparisons^a^

Outcome measure; time point	Treatment group, mean (SD) score [No. of patients]			Between-group differences					
	Empowered relief	CBT	Health education	CBT vs health education		Empowered relief vs health education		Empowered relief vs CBT	
				Estimate (SE) [95% CI]	*P* value	Estimate (SE) [95% CI]	*P* value	Estimate (SE) [one-sided 97.5% CI]	*P* value
**Pain intensity** ^**b**^									
Baseline	4.16 (1.73) [76]	4.96 (1.68) [74]	4.93 (1.59) [68]	NA	NA	NA	NA	NA	NA
Posttreatment									
1 mo	3.37 (1.88) [63]	3.40 (1.86) [57]	4.45 (2.02) [55]	−0.99 (0.29) [−1.57 to −0.41]	<.001	−0.90 (0.29) [−1.46 to −0.34]	.002	0.09 (0.28) [−∞ to 0.64]	.76
2 mo	3.08 (2.06) [61]	3.74 (2.28) [58]	4.52 (2.03) [56]	−0.88 (0.32) [−1.51 to −0.25]	<.001	−1.15 (0.32) [−1.77 to −0.53]	<.001	−0.27 (0.31) [−∞ to 0.35]	.39
3 mo	3.14 (2.02) [63]	3.20 (2.07) [60]	4.41 (1.97) [56]	−1.07 (0.30) [−1.67 to −0.47]	<.001	−1.02 (0.30) [−1.60 to −0.43]	<.001	0.05 (0.29) [−∞ to 0.63]	.86
**PROMIS pain interference** ^c^									
Baseline	58.33 (6.45) [77]	61.61 (6.06) [75]	60.83 (5.17) [68]	NA	NA	NA	NA	NA	NA
Posttreatment									
1 mo	56.12 (7.26) [63]	55.83 (7.65) [57]	58.63 (6.03) [55]	−3.37 (1.00) [−5.35 to −1.39]	<.001	−1.99 (0.98) [−3.92 to −0.06]	.04	1.38 (0.97) [−∞ to 3.29]	.15
2 mo	55.19 (7.92) [62]	55.66 (8.52) [58]	59.05 (7.01) [56]	−4.19 (1.15) [−6.46 to −1.92]	<.001	−3.19 (1.13) [−5.41 to −0.96]	.005	1.00 (1.12) [−∞ to 3.21]	.37
3 mo	54.06 (8.34) [63]	53.89 (8.65) [60]	58.85 (6.67) [57]	−4.92 (1.11) [−7.1 to −2.73]	<.001	−3.50 (1.09) [−5.65 to −1.35]	.002	1.41 (1.07) [−∞ to 3.53]	.19
**PROMIS sleep disturbance** ^d^									
Baseline	55.13 (8.22) [77]	56.20 (7.33) [75]	57.04 (6.75) [67]	NA	NA	NA	NA	NA	NA
Posttreatment									
1 mo	51.17 (9.25) [63]	53.81 (7.69) [57]	54.73 (6.69) [55]	−0.33 (1.26) [−2.81 to 2.14]	.79	−2.51 (1.23) [−4.93 to −0.09]	.04	−2.18 (1.21) [−∞ to 0.21]	.07
2 mo	49.85 (8.71) [62]	53.61 (8.29) [58]	55.47 (6.22) [56]	−1.85 (1.18) [−4.16 to 0.47]	.12	−4.64 (1.15) [−6.91 to −2.37]	<.001	−2.79 (1.14) [−∞ to 0.56]	.01
3 mo	50.01 (9.20) [63]	52.65 (9.76) [60]	57.14 (7.92) [57]	−4.15 (1.31) [−6.73 to −1.56]	.002	−5.62 (1.30) [−8.18 to −3.07]	<.001	−1.48 (1.28) [−∞ to 1.05]	.25
**Pain self-efficacy (PSEQ)** ^e^									
Baseline	39.26 (11.99) [77]	35.25 (11.33) [76]	35.49 (11.55) [68]	NA	NA	NA	NA	NA	NA
Posttreatment									
1 mo	43.37 (11.90) [63]	43.74 (13.15) [57]	38.68 (10.37) [56]	6.73 (1.45) [3.86 to 9.59]	<.001	3.73 (1.42) [0.93 to 6.52]	.009	−3.00 (1.41) [−5.78 to +∞]	.04
2 mo	44.08 (11.95) [62]	44.03 (12.65) [58]	39.52 (11.55) [56]	5.64 (1.56) [2.56 to 8.73]	<.001	3.23 (1.53) [0.21 to 6.25]	.04	−2.41 (1.52) [−5.41 to +∞]	.11
3 mo	44.54 (11.73) [63]	44.35 (13.18) [60]	37.91 (12.10) [57]	7.6 (1.62) [4.41 to 10.79]	<.001	4.68 (1.59) [1.55 to 7.82]	.004	−2.92 (1.59) [6.04 to +∞]	.07
**Pain bothersomeness** ^f^									
Baseline	4.58 (2.11) [76]	5.95 (2.25) [75]	5.69 (1.93) [67]	NA	NA	NA	NA	NA	NA
Posttreatment									
1 mo	3.43 (2.34) [60]	3.63 (2.13) [56]	5.00 (2.13) [55]	−1.25 (0.36) [−1.96 to −0.54]	<.001	−1.02 (0.35) [−1.72 to −0.33]	.004	0.22 (0.35) [−∞ to 0.92]	.52
2 mo	3.26 (2.60) [61]	3.86 (2.54) [57]	4.58 (2.36) [55]	−0.87 (0.42) [−1.69 to −0.04]	.04	−0.99 (0.41) [−1.8 to −0.18]	.02	−0.12 (0.41) [−∞ to 0.68]	.76
3 mo	3.30 (2.33) [63]	3.40 (2.57) [60]	4.86 (2.36) [57]	−1.35 (0.39) [−2.11 to −0.58]	<.001	−1.03 (0.38) [−1.78 to −0.27]	.008	0.32 (0.38) [−∞ to 1.06]	.40
**PROMIS pain behavior** ^g^									
Baseline	58.97 (3.43) [77]	59.22 (4.15) [75]	59.59 (2.87) [68]	NA	NA	NA	NA	NA	NA
Posttreatment									
1 mo	56.36 (5.36) [63]	56.38 (4.99) [57]	58.86 (3.78) [55]	−2.16 (0.73) [−3.6 to −0.73]	.003	−2.14 (0.71) [−3.54 to −0.74]	.003	0.02 (0.70) [−∞ to 1.4]	.98
2 mo	55.27 (7.21) [62]	56.61 (5.57) [58]	58.32 (4.51) [56]	−1.27 (0.90) [−3.03 to 0.5]	.16	−2.56 (0.88) [−4.3 to −0.83]	.004	−1.30 (0.87) [−∞ to 0.43]	.14
3 mo	54.58 (6.70) [63]	55.61 (6.55) [60]	58.35 (3.50) [57]	−2.08 (0.89) [−3.83 to −0.34]	.02	−2.96 (0.87) [−4.68 to −1.25]	<.001	−0.88 (0.86) [−∞ to 0.81]	.31
**PROMIS fatigue** ^h^									
Baseline	57.60 (8.05) [77]	60.46 (8.79) [75]	59.09 (6.96) [68]	NA	NA	NA	NA	NA	NA
Posttreatment									
1 mo	53.47 (10.13) [63]	55.23 (8.69) [57]	56.89 (6.53) [55]	−2.73 (1.20) [−5.1 to −0.36]	.02	−3.13 (1.17) [−5.44 to −0.82]	.008	−0.40 (1.16) [−∞ to 1.88]	.73
2 mo	53.06 (10.49) [62]	55.29 (9.45) [58]	57.22 (7.30) [56]	−3.37 (1.39) [−6.12 to −0.63]	.02	−3.74 (1.36) [−6.42 to −1.05]	.007	−0.36 (1.35) [−∞ to 2.29]	.79
3 mo	53.43 (10.93) [63]	53.01 (11.37) [60]	56.63 (7.44) [57]	−4.63 (1.48) [−7.55 to −1.72]	.002	−2.65 (1.46) [−5.52 to 0.22]	.07	1.98 (1.44) [−∞ to 4.81]	.17
**PROMIS depression** ^i^									
Baseline	53.18 (9.11) [77]	55.52 (7.88) [75]	55.23 (8.49) [67]	NA	NA	NA	NA	NA	NA
Posttreatment									
1 mo	50.78 (9.28) [61]	53.03 (8.51) [56]	54.75 (8.32) [55]	−1.91 (1.14) [−4.15 to 0.33]	.09	−1.80 (1.11) [−4.00 to 0.40]	.11	0.11 (1.10) [−∞ to 2.29]	.92
2 mo	49.29 (10.09) [62]	52.93 (9.99) [58]	54.34 (8.62) [56]	−1.74 (1.25) [−4.21 to 0.73]	.17	−2.43 (1.23) [−4.85 to 0.00]	.05	−0.69 (1.21) [−∞ to 1.70]	.57
3 mo	49.93 (9.41) [63]	52.11 (8.85) [60]	54.56 (9.04) [57]	−2.79 (1.20) [−5.17 to −0.42]	.02	−2.58 (1.19) [−4.92 to −0.25]	.03	0.21 (1.17) [−∞ to 2.51]	.86
**PROMIS anxiety** ^i^									
Baseline	54.95 (9.85) [77]	57.41 (7.42) [75]	55.51 (8.76) [67]	NA	NA	NA	NA	NA	NA
Posttreatment									
1 mo	52.40 (9.96) [61]	53.75 (8.13) [56]	54.37 (8.52) [55]	−1.60 (1.06) [−3.68 to 0.48]	.13	−1.52 (1.03) [−3.56 to 0.52]	.14	0.08 (1.03) [−∞ to 2.10]	.94
2 mo	50.91 (10.40) [62]	54.07 (9.53) [58]	53.89 (9.02) [56]	−0.82 (1.27) [−3.32 to 1.69]	.52	−2.17 (1.25) [−4.63 to 0.3]	.08	−1.35 (1.24) [−∞ to 1.09]	.28
3 mo	51.09 (9.94) [63]	52.89 (10.06) [60]	54.82 (9.49) [57]	−3.58 (1.26) [−6.07 to −1.09]	.005	−3.48 (1.24) [−5.93 to −1.03]	.006	0.10 (1.23) [−∞ to 2.51]	.93
**PROMIS physical function** ^j^									
Baseline	42.53 (6.31) [77]	40.46 (6.07) [75]	40.65 (5.46) [67]	NA	NA	NA	NA	NA	NA
Posttreatment									
1 mo	43.53 (7.51) [61]	43.26 (7.00) [56]	41.73 (5.40) [55]	1.51 (0.85) [−0.17 to 3.19]	.08	1.26 (0.84) [−0.39 to 2.9]	.13	−0.25 (0.83) [−1.88 to +∞]	.76
2 mo	43.24 (7.36) [62]	43.44 (7.43) [58]	41.65 (6.75) [56]	1.95 (0.91) [0.15 to 3.74]	.03	0.97 (0.89) [−0.79 to 2.72]	.28	−0.98 (0.88) [−2.71 to +∞]	.26
3 mo	44.25 (7.93) [63]	45.22 (7.89) [60]	41.11 (6.03) [57]	3.36 (0.80) [1.78 to 4.94]	<.001	1.83 (0.79) [0.28 to 3.39]	.02	−1.53 (0.77) [-3.06 to +∞]	.05

Abbreviations: CBT, cognitive behavioral therapy; NA, not applicable; PROMIS, Patient Reported Outcomes Measurement Information System; PSEQ, Pain Self-Efficacy Questionnaire.

^a^ *P* values are calculated using the Wald test. Analysis uses the intention-to-treat approach.

^b^ Scores range from 0 to 10 (noninferiority margin, 1.5), with higher scores indicating greater pain intensity.

^c^ Scores range from 20 to 80 (noninferiority margin, 4.0), with higher scores indicating greater pain interference.

^d^ Scores range from 20 to 80 (noninferiority margin, 1.5), with higher scores indicating greater sleep disturbance.

^e^ Scores range from 0 to 60 (noninferiority margin, 5.5), with higher scores indicating greater pain self-efficacy.

^f^ Scores range from 0 to 10 (noninferiority margin, 1.5), with higher scores indicating greater pain bothersomeness.

^g^ Scores range from 0 to 10 (noninferiority margin, 5.0), with higher scores indicating greater pain behavior.

^h^ Scores range from 20 to 80 (noninferiority margin, 4.0), with higher scores indicating greater fatigue.

^i^ Scores range from 20 to 80 (noninferiority margin, 3.0), with higher scores indicating greater anxiety or depression.

^j^ Scores range from 20 to 80 (noninferiority margin, 2.0), with higher scores indicating greater physical function.

All serious adverse events occurring during the study period (n = 9) were medical and unrelated to the study. Attrition was low after treatment initiation (empowered relief, 5.4% [4 of 74]; CBT, 7.1% [5 of 70]; and health education, 4.8% [3 of 63]). For CBT, 15.7% (11 of 70 participants) did not complete treatment (<5 sessions received). There was no statistically significant difference in baseline characteristics between participants who dropped out before treatment and those who completed month 3 surveys.

## Discussion

Our 3-arm randomized clinical comparative efficacy trial compared (1) a 2-hour pain relief skills class (empowered relief), (2) a 2-hour back pain health education class (no skills), and (3) 16-hour, 8-session group CBT in 263 adults with CLBP. The primary outcome was the between-group difference in pain catastrophizing score at 3 months after treatment. Pain catastrophizing scores at 3 months after treatment for the empowered relief group were noninferior to those of the 8-week CBT group based on a preestablished noninferiority margin of 4.3 points that was more stringent than the literature margin of 6.8^40^ and applying an α of .025. The results suggest that empowered relief effectively improves pain-related discomfort and cognitive and emotional coping.

The empowered relief group had superior pain catastrophizing scores at 3 months compared with the health education group. Similarly, for the CBT group, pain catastrophizing scores at 3 months after treatment were superior to those for the health education group, of moderate clinical importance (10.94 points in the CBT group, a difference of 48.4% from the baseline level) and established assay sensitivity. For context, multidisciplinary pain rehabilitation research has shown that a difference in pain catastrophizing score of 38% is clinically important and is associated with less disability and work status 1 year later.^41^

Empowered relief was also noninferior to CBT at 3 months after treatment for pain intensity and pain interference with correction applied for multiple comparisons for these 2 priority secondary outcomes. Empowered relief was noninferior to CBT for sleep disturbance, pain bothersomeness, depression, and anxiety, but not for fatigue and self-efficacy. Empowered relief was inferior to CBT for physical function. Minimally important difference thresholds suggest clinically important benefit across multiple outcomes for empowered relief. Although CBT is the criterion standard behavioral pain treatment, 16 hours of treatment time and associated costs can make CBT infeasible. Our results suggest that empowered relief can achieve similar results across key outcomes at 3 months.

We underscore that empowered relief is not meant to replace the longer-course CBT, which offers extended therapist contact, peer support, and didactic content (eg, functional goal setting and mood management). Rather, a range of behavioral treatment options is needed to meet the diverse needs and wants of patients. Empowered relief may improve patient access and engagement because it is adaptable to medical or community settings and may be offered at low or no cost. Empowered relief is suitable to online delivery, although efficacy may vary.

### Strengths and Limitations

Key strengths of this study bear mention. First, we applied an α of .025 for the noninferiority test (vs .05) and a pain catastrophizing score noninferiority margin of 4.3, which is more stringent than the reported 6.8.^40^ Second, we applied correction for multiple comparisons for our 2 priority secondary outcomes. Third, we applied minimal exclusions to include real-world individuals. Indeed, 64.6% had CLBP for 5 years or more, and 48.3% had 1 or more comorbid pain conditions (18.3% had ≥2). Fourth, our methods minimized regression to the mean. Last, we studied a treatment that may improve access to effective pain care. In 2019, the US Department of Health and Human Services cited empowered relief as a promising scalable pain treatment.^42^ This report provides the first early evidence, to our knowledge, that empowered relief may efficiently reduce the burden of CLBP and improve symptom management.

Several substantial limitations merit consideration. First, although our primary outcome, pain catastrophizing, is a known primary mediator of pain and function, it is less directly important for most patients and clinicians. Second, we used self-reported outcomes with unblinded interventions. Third, the study sample consisted mainly of White individuals and those who were highly educated and of higher socioeconomic status; therefore, our results may not generalize to participants with lower socioeconomic status or racially/ethnically diverse populations. Fourth, we studied CLBP, and our results may not generalize to other pain conditions. Fifth, we had substantial pretreatment attrition (approximately 20%), and the health education group had almost double the pretreatment attrition as empowered relief (empowered relief, 14.9%; CBT, 20.5%; health education, 28.4%). Although we cannot rule out disappointment with the health education group assignment, which would bias against the active treatments in per protocol analysis, we found group equivalence in treatment expectancies. Correction for multiple comparison was not applied to the nonpriority secondary outcomes, and the risk for false-positive findings exists. Last, the study was performed at a single site, all data were self-reported, and we did not control for receipt of medical care.

## Conclusions

In this randomized clinical trial of adults with CLBP, a single-session pain relief class was noninferior to 8-session CBT for pain catastrophizing, pain intensity, and pain interference and other secondary outcomes at 3 months after treatment. Future effectiveness research should include diverse patients and pain conditions, test online delivery, and address pragmatic integration into primary care.

## Notes

Supplement 1.
Trial Protocol

Supplement 2. **eTable 1.** Between-Group Differences for Posttreatment Pain Catastrophizing Scale Scores (Per Protocol) **eTable 2.** Minimally Important Difference (MID) for Secondary Outcomes **eTable 3.** Secondary Outcomes at Baseline and Posttreatment Months 1-3 by Treatment Group With Between-Group Comparisons (Intention to Treat; Adjusted for Additional Baseline Covariates)
eReferences

Supplement 3.
Data Sharing Statement
