# Clarification of Conflict of Interest Disclosure

**DOI:** 10.1001/jamanetworkopen.2022.9739

**Published:** 2022-04-06

In the Original Investigation titled “Comparison of a Single-Session Pain Management Skills Intervention With a Single-Session Health Education Intervention and 8 Sessions of Cognitive Behavioral Therapy in Adults With Chronic Low Back Pain: A Randomized Clinical Trial,”^1^ published August 16, 2021, the conflict of interest disclosure statement for Dr Mackey should have read as follows: “Dr Mackey reported receiving research funding from the NIH, US Food and Drug Administration, and PCORI (administered through Stanford University); being an unpaid advisor to ACCTION (Analgesic, Anesthetic, and Addiction Clinical Trial Translations, Innovations, Opportunities, and Networks) on their oversight committee; serving as president of the American Academy of Pain Medicine from 2014 to 2015, as a member of the American Society of Anesthesiologists Pain Committee from 2008 to 2019, and as a member of the advisory board of the American Chronic Pain Association (a nonprofit organization focused on patient education about pain), and reported receiving no compensation from these entities; and receiving travel reimbursement from the American Academy of Pain Medicine, Tarsus Group, Neurovations, International Neuromodulation Society, and FDA-ACCTION to present pain research findings.” This article has been corrected.^1^
