# Supplementary material for: Comparison of a Single-Session Pain Management Skills Intervention With a Single-Session Health Education Intervention and 8 Sessions of Cognitive Behavioral Therapy in Adults With Chronic Low Back Pain: A Randomized Clinical Trial
Source: JAMA Netw Open. 2021 Aug 16;4(8):e2113401. doi: 10.1001/jamanetworkopen.2021.13401 (PMC8369357; doi:10.1001/jamanetworkopen.2021.13401)
Supplement: Supplement 1. — Trial Protocol [file jamanetwopen-e2113401-s001.pdf]

Clinical Intervention Study Protocol

Single Session Pain Catastrophizing Treatment: Comparative Efficacy & Mechanisms

Principal Investigators:

Beth Darnall, PhD – Stanford University School of Medicine

Sean Mackey, MD, PhD – Stanford University School of Medicine

Supported by:
The National Center of Complementary and Alternative Medicine

R01 AT008561

Study Intervention Provided by:

N/A

Tool Revision History

Version Number: 7.1
Version Date: January 27th 2019

TABLE OF CONTENTS

Page

Study Team Roster **8**

Participating Study Sites **9**

Précis **9**

Study Title 9

Objectives 9

Design and Outcomes 10

Interventions and Duration 10

Sample Size and Population 12

1. STUDY OBJECTIVES **13**

1.1 Primary Objective 13

1.2 Secondary Objectives 13

2. BACKGROUND AND RATIONALE **14**

2.1 Background on Condition, Disease, or Other Primary Study Focus 14

2.2 Study Rationale 15

3. STUDY DESIGN **23**

4. SELECTION AND ENROLLMENT OF PARTICIPANTS **25**

4.1 Inclusion Criteria 25

4.2 Exclusion Criteria 25

4.3 Study Enrollment Procedures 26

4.3.1 Recruitment 26

4.3.2 Randomization 27

5. STUDY INTERVENTIONS **27**

5.1 Interventions, Administration, and Duration 27

5.2 Handling of Study Interventions 27

5.3 Concomitant Interventions 28

5.3.1 Allowed Interventions 28

5.3.2 Required Interventions 28

5.3.3 Prohibited Interventions 28

5.4 Adherence Assessment 28

5.4.1 Protocol Adherence and Treatment Fidelity 28

5.4.2 Daily Questions and Weekly Check-ins 28

5.4.3 Treatment Attendance ... 29
5.4.4 Measures to Promote Adherence ... 29
5.4.5 Specific Triggers and Stopping Rules ... 29
6. STUDY PROCEDURES ... 31
6.1 Schedule of Evaluations ... 31
6.2 Description of Evaluations ... 33
6.2.1 Screening Evaluation ... 33
6.2.2 Enrollment, Baseline, and/or Randomization ... 35
6.2.3 Blinding ... 35
6.2.4 Treatment Visits ... 36
6.2.5 Assessments ... 38
6.2.6 Study Completion ... 39
7. SAFETY ASSESSMENTS ... 39
7.1 Specification of Safety Parameters ... 40
7.2 Methods and Timing for Assessing, Recording, and Analyzing Safety Parameters ... 40
7.3 Adverse Events and Serious Adverse Events ... 40
7.4 Reporting Procedures ... 40
7.5 Follow-up for Adverse Events ... 40
7.6 Safety Monitoring ... 40
8. INTERVENTION DISCONTINUATION ... 41
9. STATISTICAL CONSIDERATIONS ... 41
9.1 General Design Issues ... 41
9.2 Sample Size and Randomization ... 41
9.3 Definition of Populations ... 42
9.4 Interim Analyses and Stopping Rules ... 43
9.5 Outcomes ... 43
9.5.1 Primary Outcome ... 43
9.5.2 Secondary Outcomes ... 45
9.6 Data Analyses ... 45
10. DATA COLLECTION AND QUALITY ASSURANCE ... 46
10.1 Data Collection Forms ... 46
10.2 Data Management ... 46
10.3 Quality Assurance ... 46

10.3.1 Training 46
10.3.2 Quality Control Committee 47
10.3.3 Metrics 47
10.3.4 Protocol Deviations 47
10.3.5 Monitoring 47
11. PARTICIPANT RIGHTS AND CONFIDENTIALITY 48
11.1 Institutional Review Board (IRB) Review 48
11.2 Informed Consent Forms 48
11.3 Participant Confidentiality 48
11.4 Study Discontinuation 49
12. SUPPLEMENTAL PROTOCOL **.......... 50**
12.1 Rationale for Supplemental Protocol 50
12.2 Objective 50
12.3 Study Design 51
13. COMMITTEES 49
14. PUBLICATION OF RESEARCH FINDINGS 51
15. REFERENCES 51
16. SUPPLEMENTS/APPENDICES 56

Appendix 1 – Schedule of Events
Appendix 2 – Informed Consent Form Template
Appendix 3 – CBT Manual
Appendix 4 – CBT Workbook
Appendix 5 – CBT Fidelity Rating Sheets
Appendix 6 – FCR PowerPoint Slides
Appendix 7 – FCR Handouts
Appendix 8 – FCR App
Appendix 9 – FCR Fidelity Rating Sheet
Appendix 10 – HE PowerPoint Slides
Appendix 11 – HE Fidelity Rating Sheet
Appendix 12 – Daily PCS Manuscript Draft
Appendix 13 – DSMP
Appendix 14 – Adverse Event Form

Appendix 15 – Serious Adverse Event Form
Appendix 16 – Concomitant Medications Form
Appendix 17 – Unanticipated Problem Form
Appendix 18 – Baseline NRS Form
Appendix 19 – Consent Process Form
Appendix 20 – Demographics Form
Appendix 21 – Inclusion Exclusion Form
Appendix 22 – Medical History Form
Appendix 23 – Participant Tracking/ Randomization and Enrollment Form
Appendix 24 – Screening Visit Checklist Form
Appendix 25 – Study Completion Form
Appendix 26 - Participant Compensation Scheme
Appendix 27 - Anuradha Roy's CV
Appendix 28 – Valerie Jackson's CV
Appendix 29 – Sue Gritzner's CV
Appendix 30 – Maisa Ziadni's CV
Appendix 31 – Kristen Slater's CV
Appendix 32 – Dennis Keane's CV
Appendix 33 – Shannon Fish's CV
Appendix 34 – Lu Tian's Biosketch
Appendix 35 - Dokyoung (Sophia) You's CV

Study Team Roster

Name	Organization	Role on Project	Phone	Email Address	Word Address
Mackey, Sean	Stanford University	Co-PI	(650)728-6477	smackey@stanford.edu	1070 Arastradero Road, Suite 200, Palo Alto, CA 94304
Darnall, Beth	Stanford University	Co-PI	(650) 721-2104	bdarnall@stanford.edu	1070 Arastradero Road, Suite 200, Palo Alto, CA 94304
Kao, Ming-Chih	Stanford University	Co-Inv	(650)723-6238	mckao@stanford.edu	Pain Management Clinic, 450 Broadway St, Pavilion A, 1st fl, Redwood City, CA 94063
Lorig, Kate	Stanford University	Co-Inv	(650)723-7935	lorig@stanford.edu	100 Welch Road #204, Stanford, CA 94305
Cook, Karon	Northwestern University	Co-Inv	(312)503-3303	Karon.cook@northwestern.edu	633 N. St. Clair, 19th Floor Chicago IL, 60611
Burns, John	Rush University Medical Center	Co-Inv	(312)942-5932	John_burns@rush.edu	1645 W. Jackson Blvd. Suite 400 Chicago, IL 60612
Sturgeon, John	Stanford University	Co-Inv/ Consultant	(650)724-8783	jasturge@stanford.edu	1070 Arastradero Road, Suite 200, Palo Alto, CA 94304
Roy, Anuradha	Stanford University	Coordinator	(650) 724-2811	anuroy@stanford.edu	1070 Arastradero Road, Suite 200, Palo Alto, CA 94304

King, Heather	Stanford University	Therapist	(650)723-6238	hking@stanford.edu	Pain Management Clinic, 450 Broadway St, Pavilion A, 1st fl, Redwood City, CA 94063
You, Dokyoung (Sophia)	Stanford University	Post-Doctoral Fellow	TBD	TBD	TBD
Tain, Lu	Stanford University	Statistician	650.721.2095	lutian@stanford.edu	HRP Redwood Building<br>Stanford, California 94305

Participating Study Sites

Stanford Systems Neuroscience and Pain Lab (SNAPL)
Stanford Center for Complementary and Alternative Medicine for Chronic Lower Back Pain
1070 Arastradero Rd, Suite 200
Palo Alto, CA 94304

Stanford Pain Management Center
450 Broadway St, Redwood City, CA 94063

Précis

Study Title

Single Session Pain Catastrophizing Treatment: Comparative Efficacy & Mechanisms

Objectives

Aim 1: Implement a comparative efficacy trial of "From Catastrophizing to Recovery" (FCR)

Aim 2: Characterize the mechanistic influence of Daily Pain Catastrophizing (PC) on future pain, function, and Trait PC

Design and Outcomes

Our design is a randomized 3-arm study comprised of 2 active psychological treatments and a health education (HE) arm that controls for time and attention. Our goals are to provide scientific evidence to demonstrate the efficacy of FCR, and also provide a comparison of said efficacy against the standard of care – group pain-CBT. Treatment allocation is randomized to minimize confounder effects. Statisticians performing analyses will be blinded.

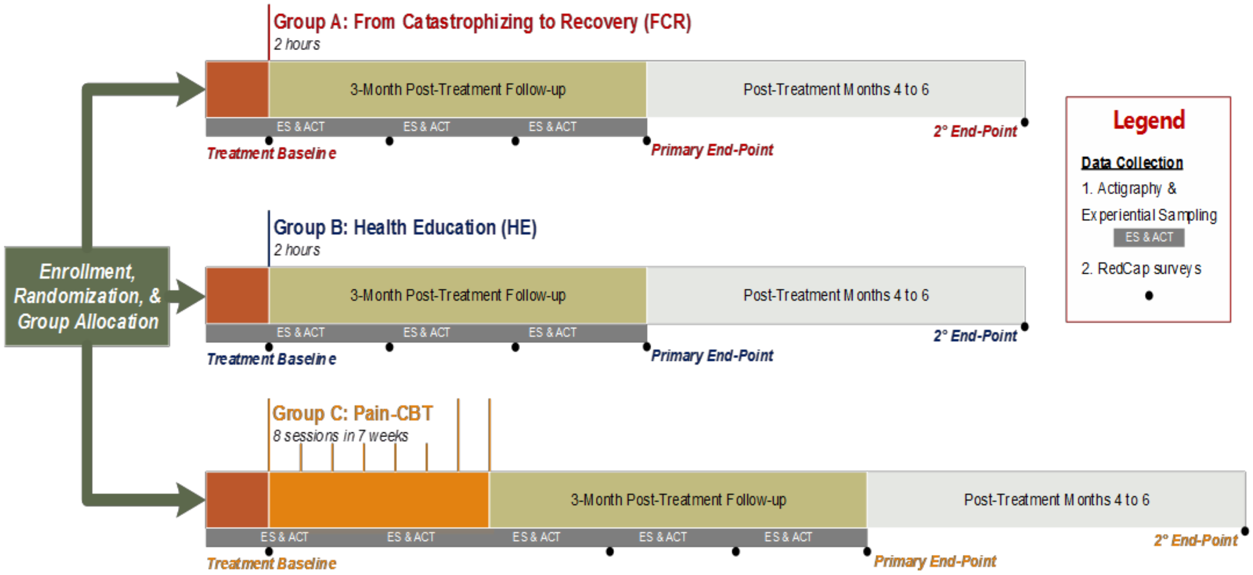

Interventions and Duration

Participants will be assessed at pre-treatment, treatment class baseline, post-treatment, and at 1, 2, 3, and 6 months follow-up.

Screening Assessment: (~2 hours – in person)

Participants will be consented for screening and will be screened for eligibility using questionnaires and the Mini International Neuropsychiatric Interview (MINI) 7.0 assessment. If eligible, participants will be consented for the study and randomized to treatment.

Pre-Treatment Assessment: (~30-60 minutes - online)

Participants will be administered a battery of questionnaires that assess pain symptoms, emotional functioning, and general health and well-being. Participants will be sent their pre-treatment assessments up to 3 days before starting treatment.

Actigraphy and Experiential Sampling (~3 minutes daily)

All participants will complete daily PCS [84] and daily pain measures online. FCR participants will answers daily skills use questions. Participants in all three groups will wear the actigraphy unit and complete daily measures for two-week periods at each of the following time-points:

> Daily Measures: All treatments will get the daily measures at pre-treatment, mid-treatment (for 8-week CBT), post-treatment, and at 1, 2, and 3 months post-treatment.

Actigraphy Unit

CBT treatment: Pre-treatment, mid-treatment (for 8-week CBT), post-treatment and 3 months post-treatment

FCR or HE treatments: Pre-treatment and post-treatment, and 3 months post-treatment

Mid-Treatment Assessment – CBT Only (~30 minutes - online)

Participants will complete online questionnaires that assess pain symptoms, emotional functioning, and general health and well-being.

0, 1, 2, 3, 5* (*for FCR and HE), and 6-month Post-Treatment Assessments (~30-60 minutes - online)
Participants will complete online follow-up questionnaires that assess pain symptoms, emotional functioning, and general health and well-being.

Note: All post-treatment surveys must be completed within 3 weeks following each post-treatment time point. We have multiple systems in place to ensure data is collected within days of survey deployment. Our survey system will close and lock 3 weeks following survey. All data will be time-stamped in the event NCCIH requires a smaller window.

Interventions

Group A. "**From Catastrophizing to Recovery (FCR)":** A single-session approximately 2-hr group intervention to treat PC.
Format of intervention: Therapist-delivered PowerPoint presentation with experiential exercises. Experienced senior level psychologist and two (2) additional doctoral level psychologists will conduct the FCR sessions. Respectively, the instructors include Drs. Valerie Jackson, Kristen Slater, and Maisa Ziadni.
Content of intervention: See Preliminary Study 1 (2014, *J Pain Res*). FCR Participants have the **'FCR** Relaxation Resource' app (guided relaxation audiofile) loaded on their apple or android electronic device. Participants will be given an electronic tablet for the duration of the study if they do not own an appropriate device.

Group B. Health Education (HE) Intervention: We aim to control for the non-specific effects of (1) receiving a 'treatment', (2) participating in a research study, and (3) providing daily ratings for PC and pain. The 2-hour HE group will match FCR on 4 important factors: duration, structure, format and site [46].
Format of intervention: The HE class will be expert-led by doctoral level health educator, Kate Lorig, DrPH, as well as 2 additional and will involve a PowerPoint presentation and handouts. Two additional providers will be identified and trained to conduct the sessions. Selected instructors are Dennis Keane and Shannon Fish; both individuals have substantial health education experience.
Content of intervention: HE content will include basic information such as nutrition and interacting with the healthcare system; these relatively 'inert' topics are unlikely to impart any specific effects typically found with a true psychobehavioral intervention [46].

Group C. 8-week Manualized Pain-CBT Group Intervention: We will test non-inferiority of FCR compared to the current gold standard group treatment for PC-- manualized 8-session pain-CBT.
Format of intervention: Group CBT will be delivered by PhD-level psychotherapists (3 in total) under the supervision of Beth Darnall, PhD, who has >10 years experience in the delivery of CBT for pain. The group is run weekly for 8 consecutive classes. Each class is 2 hours with a midpoint break (16 hours total intervention time). Two group cohorts may run concurrently. Groups may be offered at two

different times of day to optimize enrollment (e.g., 10am and 4pm). Instructors include: Drs. Heather King, Uta Maeda, and Sue Gritzner.
Content of intervention: The protocol and materials, including the therapist manual [47] and patient workbook [48], were developed with funding from NIH/NCCAM R01 AT006226 (Dan Cherkin, PI), and include content from a pain-CBT program developed by Drs. Ehde, Dillworth, and Turner [49]. This specific manualized CBT treatment is also being used in the CBT arm of our NCCIH P01 treatment outcome study for CLBP with Drs. Darnall and King as the CBT therapists), thereby assuring proficiency with the content and delivery of the intervention. We will use treatment fidelity checklists developed by Dr. Cherkin's group [See Appendix 5]. The intervention content is included in full in Appendix 3. Participants receive a workbook with homework (See Appendix 4) and a copy of *The Pain Survival Guide: How to reclaim your life* © 2005 by Turk & Winter [51] for optional reading.
Treatment Completion: The threshold for pain-CBT completion is attendance at 5 of the 8 classes. Participants missing more than three (3) classes will be considered non-completers.

Sample Size and Population

The project will enroll 231 adults (ages 18-70) who meet criteria for axial low back pain without radicular symptoms. Eligibility will be assessed by the research staff. Participants will be randomized to one of the 3 treatment conditions.

1. STUDY OBJECTIVES

Our design is a randomized 3-arm study with test treatment, active control, and placebo arms. Our goals are to provide scientific evidence to demonstrate the efficacy of FCR, and also provide a comparison of said efficacy against the standard of care – group pain-CBT. Treatment allocation is randomized to minimize confounder effects. Statisticians performing analyses will be blinded.

1.1 Primary Objective:

We will conduct a 3-arm comparative efficacy RCT in 231 patients with CLBP comparing: (A) FCR, (B) 8-session group pain-CBT--the gold standard for group pain psychology--and (C) a health education (HE) control group. Our endpoints are post-treatment PC at 3 months (primary) and 6 months (secondary). Advanced analytics (machine learning) will be also be used to characterize responders and non-responders.

- Hypothesis 1a (Superiority): FCR will be superior to HE for Trait PC reduction.
- Hypothesis 1b (Assay sensitivity): 8-week CBT will be superior to HE for Trait PC reduction.
- Hypothesis 1c (Non-inferiority): FCR will be non-inferior to 8-week pain-CBT for Trait PC reduction.
- Hypothesis 1d (Global improvement): FCR will be superior to HE and non-inferior to 8-week pain-CBT for longitudinal changes in PROMIS pain intensity and behavior, fatigue, and sleep disturbance using rmANOVA.
- Hypothesis 1e (Improvement in objective measurement of function): Reduction in PC provided by FCR is associated with improvements in objective physical function as measured by actigraphy.

1.2 Secondary Objectives

We will use multi-level modeling to characterize treatment effects on Daily PC fluctuations, and the impact of Daily PC changes on Trait PC, longitudinal PROMIS measures, and actigraphy (activity and sleep).

- Hypothesis 2a (Level-1 PC effect): Daily PC will predict same-day and next-day levels of pain and activity.
- Hypothesis 2b (FCR/CBT moderation of Level-1 PC lagged effect): The relationships between daily PC and same day and next day pain and activity are reduced by FCR and CBT interventions compared to HE.
- Hypothesis 2c (Level-2 FCR effect): Daily PC mean changes (baseline to 1 month post-treatment) will predict mean change in pain, activity, sleep and Trait PC in the FCR and 8-week CBT groups at months 1-3.
- Hypothesis 2d (Level-1 FCR/CBT skills use lagged effect): Previous day and same day use of FCR / CBT skills will predict next day improvement in Daily PC.

2. BACKGROUND AND RATIONALE

2.1 Background on Condition, Disease, or Other Primary Study Focus

The Institute of Medicine (IOM) recently reported that chronic pain affects about 100 million U.S. adults and costs the nation $635 billion [11]. Chronic low back pain (CLBP) is cited as the most common type of chronic pain [11], and rates continue to rise despite incrased utilization of treatments such as surgery and pharmacology. For instance, in North Carolina the prevalence of CLBP rose from 3.9% in 1992 to 10.2% in 2006, along with disability and health care costs [1].

Clearly, there is an urgent need for effective interventions to prevent and better treat CLBP once it is established. Multiple studies suggest that the greatest predictor for the development and progression of CLBP is pain catastrophizing (PC) [3] (review). While PC is effectively treated with pain-CBT, access to care may be limited by the following common barriers: (1) need for physician referral; (2) poor reimbursement or no insurance; (3) co-payment costs and (4) travel burden, time and costs associated with the 6-10 group or individual pain-CBT treatment sessions. **This proposal addresses a critical problem in CLBP: poor access to PC treatment.**

To address this problem, we developed a unique intervention that specifically treats PC. **"From Catastrophizing to Recovery (FCR)"** is rapidly delivered in a single-session class, thereby eliminating many of the barriers associated with traditional, multi-session pain-CBT. Our pilot data show that FCR significantly reduces PC one month post-treatment, even in patients with comorbid depression and anxiety [7]. The primary goal of this application is to rigorously test FCR, a low-burden, low-cost intervention that holds promise as being effective, accessible, and broadly applicable. Therefore, this proposal **challenges and seeks to shift current clinical practice paradigms with a novel treatment approach that could transform the way PC is treated—and expand access to low-cost, non-pharmacologic CLBP care.**

<u>**The Problem of Pain Catastrophizing (PC) in Chronic Low Back Pain (CLBP):**</u> Consistently across studies, PC is a primary predictor for the onset and worsening of CLBP, even amongst all surgical and clinical variables [3,12,13]. PC is a cognitive-emotional response pattern involving negative expectation and appraisal about actual or anticipated pain and includes feelings of helplessness about pain [14]. A systematic review of PC in CLBP (total N = 2,269) found that PC predicted pain intensity and disability, and PC mediated CLBP treatment efficacy [3]. Independent of depression, in CLBP patients PC is associated with an array of negative phenomena including increased affective distress [6], muscle and joint tenderness [4], muscular tension at rest [15], pain-related disability [4, 5], poor response to various pain treatments including surgery [6, 15, 16] [5, 17], and to greater use and misuse of opioids [18].

PC is also harmful in the context of acute pain and even for individuals who are pain-free. For instance, PC is implicated in the persistence of back pain [12] and it was shown to account for 47% of the variance in the *development* of chronic back pain following an episode of acute back pain [13]. Moreover, a population study revealed that among all factors investigated, PC best predicted the acquisition of CLBP one year after a pain-free baseline [19]. Taken together, these data suggest that PC is a major health risk for all Americans, and that early treatment for PC may serve as prophylaxis for CLBP.

Given that PC appears to undermine response to medical treatment for CLBP it is perhaps unsurprising that *reductions in PC are associated with subsequent* improvements in pain and intervention effectiveness. In a prospective study that included primarily CLBP, Burns (Consultant – See Preliminary Study 13) et al used cross-lagged analysis to show that early reductions in PC significantly improved

later response to multidisciplinary treatment in terms of pain intensity and pain interference [20].

The primary treatment for PC is psychotherapeutic intervention that is rooted in cognitive behavioral theory and specific to chronic pain (pain-CBT). Pain-CBT typically focuses on identifying and changing maladaptive cognitive (e.g., catastrophizing or negative beliefs) and behavioral (e.g., avoidance) patterns that serve to maintain or worsen the pain experience and outcomes. A key component of pain-CBT involves the acquisition and use of skills, such as problem-solving, relaxation, and thought restructuring. A recent meta-analysis of RCTs (4788 participants across 35 studies) determined that at post-treatment, pain-CBT is more effective for reducing PC than usual care or active control [21]. Research findings suggest that the efficacy of pain-CBT for treating pain and other outcomes is actually mediated by post-treatment changes in PC [6, 22, 23]. While we know the broad recipe for treating PC with pain-CBT, little is known about the individual ingredients, how they function to reduce PC, and how changes impact related variables. To fill this gap in understanding, we propose to elucidate the mechanisms of PC and PC treatment (Aim 2).

2.2 Study Rationale

Current PC Treatment Poses Patient Burdens and Barriers to Care: The most cost effective treatment for PC is 6-10 session group pain-CBT. Often referred to as coping skills classes, the content of group pain-CBT is broad and includes pacing, self-care, sleep hygiene, goal setting, communication skills, and mood management in addition to PC treatment. While pain-CBT effectively treats PC, it involves burdens associated with multiple visits, travel costs, copays, and time away from work or other obligations. Each group treatment session lasts 1.5-2 hours, and with travel the total time commitment ranges from 16-30 hours. Such burdens may prevent access to PC care, thereby undermining future pain treatment [3] and promoting use of less effective but readily accessible modalities such as medications and costly procedures.

We identified only one pain-CBT intervention developed specifically to treat PC and it was developed for chronic headache [24]. The intervention posed substantial time burden (10 sessions, 1.5 hours each, 15 hours total) and included topics peripheral to PC, such as pacing and assertive communication. The 6-month moderate effect size (Cohen's *d* = 0.53) was consistent with other pain- CBT studies (See *Cochrane Review*, [21]). The most common reason for declining to participate in the study was the 10-week time commitment for treatment [24]. Thus, the time burden *alone* may pose a formidable barrier to PC care.

We propose to study PC and PC treatment in CLBP—the most common chronic pain condition in the U.S. **Our proposal seeks to change current practice paradigms because it sets forth the concept that a targeted, single-session intervention has enduring, high-impact benefit.** The brief FCR intervention is not meant to replace longer-course pain-CBT--a modality that is proven to work well; rather, FCR delivers effective, targeted *PC treatment* that ascribes minimal burden and therefore broadens access to PC care. **Successful completion of our aims will improve clinical practice** by addressing the urgent need to develop safe, targeted, effective, low cost pain treatments that are widely accessible. In doing so, we will dismantle a **primary critical barrier to progress in the field: lack of a specific and *efficient* treatment pathway for PC**. In addition to developing FCR, we will characterize responders and non-responders to this targeted PC therapy, thus allowing for refinements that meet the needs of subpopulations.

Mechanisms of PC Treatment: Types of PC include: Trait PC, State PC, and Daily PC. State PC is situational in nature, typically is measured in evoked pain paradigms, and is not relevant to the goals of this proposal.

TRAIT PC measures dispositional catastrophizing in the context of actual or anticipated pain. Measurement: The Pain Catastrophizing Scale (PCS; 13 items) [14] and the Catastrophizing Subscale of the Coping Skills Questionnaire (CSQ; 6 items) [25] are validated measures of Trait PC that have been widely used in clinical and research settings. The PCS assesses 3 factors of PC (magnification, rumination and helplessness) and therefore is considered to be more comprehensive than the PC subscale of the CSQ, which measures helplessness and pessimism. The PCS measures the frequency of negative cognitive and emotional responses within the broad context of pain. Gaps in Knowledge: Little is known about how Trait PC changes. **We aim to fill critical gaps in knowledge regarding how daily factors (Daily PC and daily pain) influence Trait PC, and which Trait PC factor(s) are most malleable and responsive to treatment and to daily fluctuations in PC and pain.** The high frequency PC sampling we propose is critical to achieving these aims.

DAILY PC quantifies PC over the previous 24-hour time frame. Capturing Daily PC longitudinally for a specified time course (e.g. 1 month) allows for the examination of how PC adaptation / change occurs in response to treatment [26]; in other words, how PC functions as a process variable. Important mechanistic questions can also be addressed with Daily PC measurement [8, 27] including how changes in PC shape adaptation / changes in daily mood or daily pain. **Many of the current knowledge gaps for Trait PC may be filled by characterizing Daily PC and its longitudinal influence on Trait PC (our Aim 2)**. Several studies that have reported to measure "State PC" were in fact reporting Daily PC, typically using 2 or more questions from the CSQ [8]. Researchers have used the CSQ in part or whole because its brevity lends greater suitability for daily assessments. However, a major limitation of the PC subscale of the CSQ is that it has only 2 factors and therefore is not fully comparable to the PCS. Measurement: *There is no validated tool for measuring Daily PC*. Gaps in Knowledge: There is a critical need to develop a PC tool that (1) is validated for daily use and (2) measures all 3 factors of the PCS. **Our proposal will shift current research paradigms and advance PC measurement methodology by developing a validated tool for *comprehensive Daily PC assessment*.** We have validated 3- and 5-item Daily PCS measures [84]. The 5-item Daily PCS contains all items from the 3-item Daily PCS (all rumination subscale items) as well as single items from the magnification and helplessness subscales. We will use the 5-item Daily PCS measure to examine the mechanics of PC in terms of (1) how Trait PC responds to mean Daily PC fluctuations; (2) how mean Daily PC responds to the 2 pain-CBT interventions; and (3) how Daily PC relates to adaptation to pain and longitudinal changes across PROMIS domains. Our Daily PCS measure will facilitate standardized measurement for future studies that examine Daily PC, thereby improving accuracy of analytics, interpretability, cross-study comparisons, and meta-analyses. **Our proposal seeks to dismantle another barrier to progress in the field:** lack of understanding regarding PC mechanics. We will use our Daily PCS measure and daily diary data to elucidate the mechanisms that change Trait PC with and without targeted treatment. We will also characterize how Daily PC and Trait PC influence pain, mood, fatigue, behavior, sleep, and function with the context of pain-CBT and targeted treatment using subjective and objective measures.

Successful completion of our aims will significantly advance scientific knowledge in a number of ways. Even if FCR emerges as inferior to 8-session pain-CBT at a *group* level, we will significantly advance scientific understanding regarding Daily and Trait PC, response to treatment and mechanisms of change, their impact on pain and related variables. Our combination of actigraphy, daily measures, and use of PROMIS measures will allow us to characterize these relationships and PC mechanics in CLBP. We will use advanced analytic techniques (e.g., machine learning) to characterize responders and non-responders to both active interventions, thus providing comprehensive data that will elucidate the mechanisms of both treatments. Phenotyping will reveal who is likely to benefit from single-session PC treatment and future studies will focus on expanding and fast-tracking care to this responsive subpopulation. **Successful completion of our aims will alter pain treatment and *preventative***

interventions* that drive the field of pain treatment.** The efficient format of FCR will facilitate application across variety of settings--such as in primary care (e.g. acute back pain, work related injuries) or in pre-surgical populations—where PC treatment stands to *prevent the development of CLBP* [4, 5, 12]. Our group found that pre-surgical PC predicts time to opioid cessation, and others have associated PC with persistent opioid use after lumbar surgery [17]. Accessible and effective PC treatment *prior to surgery* would be **a critical and novel *preventive intervention that would reduce several factors including post-surgical acute pain, chronic pain, misuse behaviors that develop in response to untreated PC [18], and would speed time post-surgical opioid cessation.

We have developed all the experimental tools and techniques required to accomplish our specific aims. We describe here our ability to successfully: (1) Develop and test our single-session class designed to specifically treat PC, and measure its related impacts on pain and health; (2) Use our validated Daily PCS measure [84] to characterize mechanisms of PC treatments and elucidate how Trait PC changes; (4) develop and implement a novel Android ESM platform; (5) integrate this multimodal data into models using machine learning techniques to better predict responders and non-responders to FCR and 8-session group pain-CBT.

Our proposal will shift current research and clinical paradigms by developing an intervention that is highly novel because it is *targeted* and *brief* PC treatment and therefore would be a highly accessible pain treatment across conditions, patients, and settings—and, if successful—would be chronic pain prophylaxis and potentially an important tool to minimize opioid use. **The FCR intervention is highly scalable and easily lends itself to broad dissemination across settings—outpatient, inpatient, pre-surgical, and community--through various technologies** (e.g. DVD or a web based video class). We will characterize how reduced variability in Daily PC and mean levels of Daily PC influence future Trait PC, *thereby revealing the mechanisms that facilitate enduring changes in PC*. Such critical knowledge will facilitate the development of interventions that *specifically target the factors that facilitate positive cognitive and emotional neuroplasticity*. In future studies we will characterize the neural correlates of PC plasticity within the context of treatment.

We ***propose to use novel methodologies*** to achieve our aims, including our computer adaptive testing algorithm for NIH PROMIS items, CHOIR-CAT, which has advanced features for estimation, item selection, inference, and stopping conditions, thus yielding smaller standard errors of estimation (SE) while using fewer items. CHOIR-CAT is used in our open source Collaborative Health Outcomes Information Registry (CHOIR) and, compared against classic testing theory-based instruments, reduces subject burden by 71% [9, 10, 28]. We will leverage CHOIR-CAT within the REDCap system and will combine it with daily ESM and objective actigraphy data for sleep and activity to comprehensively characterize how PC—and PC changes—impact medical, and psychosocial functioning. We are aware of no studies that used actigraphy to objectively quantify how activity and sleep respond to PC reductions (though our FCR pilot data detected a signal for an inverse relationship); thus **our proposed objective data methods are novel--and critical--because they eliminate potential response bias regarding symptoms and behavior**. Additionally, we will refine our Android app (FCR Relaxation Resource) and reduce response bias by objectively quantifying use of the FCR autogenic / relaxation audiofile, thus providing a clear marker of behavior in one's own environment, treatment response and 'compliance'. Finally, our proposed ESM system will allow for real-time compliance checking, and thus will reduce missing data and will bolster our power to detect treatment effects using ITT analytics.

Preliminary Studies

Preliminary Study 1: "From Catastrophizing to Recovery" (FCR) Intervention was developed at the Stanford Systems Neuroscience and Pain Laboratory (2014, *J Pain Res*) [7]. FCR has 2 main components: education and skills acquisition. First, participants learn mind-body science as it relates to pain and PC. They learn to identify PC and how to self-treat it. Self-treatment involves applying skills to decrease physiological hyperarousal--diaphragmatic breathing and progressive muscle relaxation-- within the context of PC. Self-treatment also involves applying skills that improve cognitive and emotional regulation, including PC reframing and thought restructuring. Participants identify their typical PC thoughts and practice writing out personal reframes. Finally, self-treatment includes enacting behaviors that modulate attention and counteract helplessness. Participants self-tailor the information by developing a comprehensive plan to stop and prevent PC (See Appendix 7). Participants leave the class with the following tangibles: (1) their own written, self-crafted, personalized PC cessation plan; (2) a 20-minute relaxation response audio CD; and (3) a printed copy of the FCR didactic content to access as needed in their PC cessation plan.

We delivered FCR to 76 mixed etiology patients with chronic pain at the Stanford Pain Management Center who were referred by a treating provider. The PCS was administered at class check- in (baseline) and post-class at 2 and 4 weeks electronically. Class size was 4-18 participants. The class was delivered as free clinical care and without compensation for completing two PCS follow-up measures.

**Table C1. Post-Class Survey Results**	0-6	
**Item**	**Mean**	**Percentage**
**How acceptable did you find this class?**	5.6	Acceptability: 94%
**How satisfied are you with this class?**	5.5	Satisfaction: 92%
**How useful was the information presented?**	5.6	Usefulness: 94%
**Was the information easy to understand?**	5.7	Ease of Understanding: 95%
**How likely are you to use the skills and information you learned today?**	5.5	Likelihood of Using Skills and Information Learned: 92%

**Table C2. PCS by time point**	
**Time point**	**Mean (SD)**
**Baseline**	25.47 (10.71)
**Week 2**	16.47 (10.52)
**Week 4**	13.82 (9.5)

Feasibility: We demonstrated feasibility for enrollment and successful delivery of FCR across 13 class cohorts, collection of pre-post treatment data, and use of PROMIS longitudinal outcomes (Preliminary Study 2). High ratings for the 5-item post-class anonymous survey (0=very dissatisfied, 6=very satisfied) suggest that FCR has good face and content validity, and that it is well-received (Table C1).

We found no differences in baseline PCS scores between those who did or did not complete follow-up questionnaires. Data included 51 participants who completed at least one follow up PCS: 67% of the full sample; Week 2, n=43 (84%); Week 4, n=34 (67%). (NB: Some participants completed only one follow-up PCS). Mean age was 49.95 (SD = 12.14) and 82% female. While patients with psychologist diagnosed depression (64%) and anxiety (70%) had higher baseline PCS scores we found no difference in absolute treatment response compared to those without the comorbidities.

Clinical Significance and Effect Sizes for FCR: We used within subjects rmANOVA with time as the within-subjects factor and anxiety and depression as between subjects' factors. We found a significant linear effect for time indicating significantly reduced PCS scores at weeks 2 and 4 compared to baseline ($F = 45.97$, $p<.001$). Post-hoc paired t-tests revealed significant within subjects differences for all contrasts: PCS Baseline to PCS Week 2 ($N = 43$; $t = 6.07$, $p < 0.001$); PCS Baseline to PCS Week 4 ($N = 34$; $t = 8.36$, $p < 0.001$); and PCS Week 2 to PCS Week 4 ($N = 24$; $t = 2.51$, $p = 0.02$). Effect sizes were large for baseline to 2 and 4 week follow-up (Cohen's $d = 0.85$ and Cohen's $d = 1.15$, respectively). Importantly, these effects sizes are similar or larger than effect sizes for PC reductions reported for standard 8-session CBT- based classes [21]. While impressive, it is important to note that our study was uncontrolled and there was significant drop out inherent in the design. Accordingly, we did not use this estimate to power our study. Instead we conservatively derived anticipated effects from studies in the literature which were controlled and had various adjustments for drop-outs and missing data. Most PCS score changes in our pilot study met the IMMPACT threshold for a clinically meaningful improvement [29]. Baseline PCS did not predict treatment response—similar to other pain-CBT interventions [23]—thus suggesting that FCR may be broadly appropriate for people with chronic pain. Our data suggest that treatment effects may strengthen over time with ongoing use of the personalized plan.

Relevance: FCR was feasible, broadly acceptable, and significantly reduced PC at 2 and 4 weeks post-treatment. We aim to build on these promising data by conducting a rigorous comparative efficacy RCT. Preliminary data support our proposed methods to phenotype responders and non-responders.

Preliminary Study 2: Effect of FCR on NIH PROMIS Assessments Controlling for 'Treatment as Usual'

At each visit to the Stanford Pain Management Center (SPMC) patients complete a battery of pain and psychosocial measures--including computerized automated testing (CAT) versions of the NIH PROMIS measures through the use of CHOIR (Collaborative Health Outcomes Information Registry; funded through NIH Pain Consortium HHSN 271201200728P). We examined the impact of FCR across multiple PROMIS domains. 21 FCR participants had CHOIR data for the following two time points: (1) 1 month pre-FCR and (2) 1-3 months post-FCR. (N.B. The remaining participants had no CHOIR data due to stopping treatment at the Pain Clinic. We now capture CHOIR longitudinal data from patients after clinic discharge.) Paired t-tests showed positive pre- to post-FCR changes for PROMIS Pain Intensity, Pain Interference, Depression, Sleep Disturbance, and Function ($p < 0.001$). To control for 'treatment as usual' effects we compared a clinic cohort matched to FCR participants on sex, age, and PCS scores. The FCR

**Table C3: Pain Catastrophizing Scale (PCS) Outcomes**				
**Clinical Significance**	**Week 2**		Week 4	
	N	PCS Change From Baseline	N	PCS Change From Baseline
**Increased PCS**	4	+ 20.3%	0	
**No Change**	2	- 5.5%	3	-9.0%
**Minimally Important**	12	- 22.6%	5	-25.0%
**Moderately Important**	12	- 40.4 %	7	-38.9%
**Substantially Important**	13	- 61.5%	11	-72.4%

group evidenced superior improvement for PROMIS Pain Interference (Cohen's d = 0.73, p < 0.001), Pain Behavior (Cohen's d = 0.66, p < 0.001), Fatigue (Cohen's d = 0.26, p = 0.05), and Sleep Disturbance (Cohen's d=0.46, p < 0.001). Note an advantage of the NIH PROMIS is that by construction, PROMIS score effect sizes can also be estimated against the reference Census 2000 population by dividing the mean difference by 10.

Relevance: **Our data, while preliminary, suggest that the positive impacts of FCR extend beyond PC--and usual care--and include multiple pain and health domains.** These data establish a rationale for the specific objective data (actigraphy for sleep and activity) in the proposed study and establish our ability to leverage PROMIS.

Preliminary Study 3: Effect of FCR on NIH PROMIS Assessments in the Research Setting

We enrolled participants in a second FCR study and extended measurement to 8 weeks post-FCR. This pilot was designed to mimic our proposed study and to (1) assess feasibility, (2) provide week 8 data, and (3) measure PROMIS pain outcomes at standardized time points.14 subjects completed measures at baseline and 4 and 8 week follow-up. One-way rmANOVA was used, and post-hoc paired t-tests were used with adjusted p-values. Given the small sample size, Friedman's test was also used, with similar results. We found reductions in PCS (p<0.001, Cohen's d=0.57 at 4 weeks and 0.76 at 8 weeks) and PROMIS Pain Interference (p=0.013, Cohen's d=0.85 at 8-weeks). Wilcoxon signed rank test showed PROMIS Pain Intensity at 8 weeks was reduced (p= 0.014, Cohen's d=0.63).

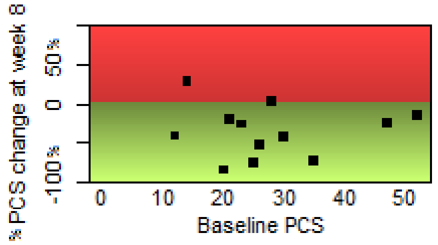

Relevance: **While small and uncontrolled, pilot data signal enduring PCS reduction and reduction in pain variables.**

Preliminary Study 4: Experiential Sampling Methods & Influence of State Catastrophizing

See our publication (2013, Annals of Behavioral Medicine). Our group examined the influence of Daily and Trait PC in 231 adults with chronic pain using daily ESM [8]. Daily PC was found to account for a significant proportion of the relationship between daily pain, affect, and mood. Greater Trait PC was found to amplify the effect of Daily PC on pain and emotional states. **Importantly, we administered daily a portion of the CSQ catastrophizing subscale as there was no validated tool available to assess Daily PC.**

Relevance: This study demonstrates our experience with daily ESM and analytics, proficiency with time-lagged modeling, and illustrates the pressing need to validate a Daily PC measure to elucidate PC mechanics.

Preliminary Studies 5-7: Daily Electronic Sampling Methods (ESM) in Chronic Pain Clinical Trials

We have conducted 3 clinical trials using ESM. Our first 2 studies examined daily pain and symptom ratings in women with fibromyalgia and the effect of a novel glial cell modulator (low dose naltrexone) (See our publications Arthritis & Rheum, 2012; Pain Med, 2009) [36, 37] Our third study characterized the influence of PC on negative and positive affect in 260 women with chronic pain (Pain, 2013) [38]. We characterized the relationships between stable and day-to-day fluctuations in PC, negative and positive affect, pain intensity and positive and negative interpersonal events using daily ESM across 30 days. Daily PC significantly mediated the relationship between pain intensity and positive and negative affect, in both stable and daily-variance models.

Relevance: Further illustration of our expertise in ESM, Daily PC measurement, and mediation analytics of hierarchical datasets in clinical trials to characterize outcomes. Findings elucidate Daily PC impact on

mood and support our plan to validate and use our 3-factor Daily PC measure with broad behavior assessment.

Preliminary Study 8: We developed a 3-Factor Brief PCS

In preliminary work we conducted confirmatory factor analysis on PCS scores for 607 clinic patients. The original PCS validation yielded 3 factors (magnification, rumination, and helplessness) [14], also confirmed by others [30-32]. As the 3 factors are theoretically and statistically correlated, we specified a 3-factor structure, allowing for correlation between factors. The 2 items that correlated highest with each latent factor were combined to create the 6-item brief measure.

Relevance: Building on this initial work we **developed and validated a Daily PCS** measure in months 1-6 using independent chronic pain samples [84]. A validated Daily PCS will enable us to characterize fluctuations in Daily PC —within the 3 subscales. The daily diary PC data are critical to elucidating how changes in daily PC lead to changes in other variables, and ultimately into lasting treatment effects.

Preliminary Study 9: RCT Experiment Shows State PC Predicts Cytokine Response

Based on the results of our previous work [33], our group (Darnall and Mackey) conducted an RCT to examine the influence of State PC [34] and Trait PC [35] on response to a PC induction experiment in women with chronic pain. For the experiment group (PC induction), we found that greater State PC experienced during the induction predicted significant increases in IL-8 and TNF-a for post-menopausal women only (N = 34) and not for pre-menopausal women (N = 46); no effect was found for the control group).

Relevance: We found that State PC – not Trait PC—was associated with pro-inflammatory cytokine response in post-menopausal women; findings show a link between PC and immune response. Our findings highlight the role of state/daily PC biological responses relevant to chronic pain – and the importance of stopping and preventing PC.

Preliminary Studies 10-12: Proficiency with Machine Learning Techniques

Studies by our group (PLOS One, 2011; Cerebral Cortex, 2012) used machine learning analysis techniques, including supervised learning with support vector machines and algorithms for the purpose of data pattern recognition, classification and regression analysis in acute and CLBP [39, 40]. Additional studies used large-scale multivariate regression framework and unsupervised machine learning such as hierarchical clustering to adjust actigraphy data and discover unique signatures of alteration in physical activities [41].

Relevance: We have demonstrated competency with the advanced statistical methods we will use to develop prediction models to phenotype and determine treatment responders and non-responders.

Preliminary Study 13: Early Reductions in PC Lead to Improved Response to Multidisciplinary Care

Dr. Burns (Consultant) and colleagues (J Consul Clin Psychol, 2003) used cross-lagged panel analysis to show that (a) early-treatment PC reduction predicted late-treatment response to pain care, but not vice versa; and (b) these effects remained significant with depression change controlled [20, 22]. Findings provide a foundation for our Aim 2 hypotheses, and illustrate our team's experience with the time series PC analytics.

Preliminary Study 14: PC Mechanisms of CBT Therapy for Chronic Pain (Transl Behav Med, 2012) This study illustrates (1) our group's experience with examining PC mechanisms within the context of an RCT, (2) PC mechanics related to pain-CBT, and (3) methodological considerations regarding PC mechanics [42].

PRELIMINARY STUDIES SUMMARY: Our preliminary data provide a strong rationale and foundation of expertise to support our proposed scientific aims and methodology. Our pilot data evidence feasibility, and good participant satisfaction and understandability ratings for FCR. We found enduring PC reductions and improvements in pain and health outcomes compared to a clinic control cohort. We have assembled a specialized team of experts who address the key methodological aspects of the current proposal: measuring Daily PC, ESM and analytics, actigraphy, machine learning analytics, cross-lagged panel analysis to characterize PC mechanics, and RCTs in chronic pain. We have a strong history of productive research collaboration, successful completion of projects, and excellent capacity to leverage existing resources.

3. STUDY DESIGN

Our proposed study is a parallel-groups, -time and attention-controlled, triple-blind, combined superiority and inferiority trial of a novel brief intervention to treat pain catastrophizing. Our nationally respected research team includes:

- **Sean Mackey, MD, PhD**, as leading expert on research on CLBP, expertise in the conduct of pain RCTs, use of ESM, CHOIR and PROMIS in chronic pain research.
- **Beth Darnall, PhD**, with expertise in catastrophizing research, intervention development, and the conduct of RCTs involving psychobehavioral interventions for CLBP.
- **Kate Lorig, RN, DrPH** as national expert in the development and validation of measurement tools for pain populations, in patient education, and the conduct of large-scale, and longitudinal RCTs.
- **Karon Cook, PhD** as national expert psychometrician in scale development, validation, and assessment; methodology and application of PROMIS in chronic pain research.
- **John Burns, PhD** with expertise in research methods and analytics for pain catastrophizing and the use of time series, cross-lagged analyses within the context of RCT treatment-outcome studies.
- **John Sturgeon, PhD** with expertise in Daily PC measurement and ESM analytics.
- **Heather King, PhD** is an expert in group pain-CBT.
- **Ming-Chih Kao, PhD, MD** with expertise in biostatistics, RCT analytics, actigraphy, ESM and analytics, App and database development, large data management, and machine learning techniques.

Our design is a randomized 3-arm study with test treatment, active control, and placebo arms. Our goals are to provide scientific evidence to demonstrate the efficacy of FCR, and also provide a comparison of said efficacy against the standard of care – group pain-CBT. Treatment allocation is randomized to minimize confounder effects. Statisticians performing analyses will be blinded.

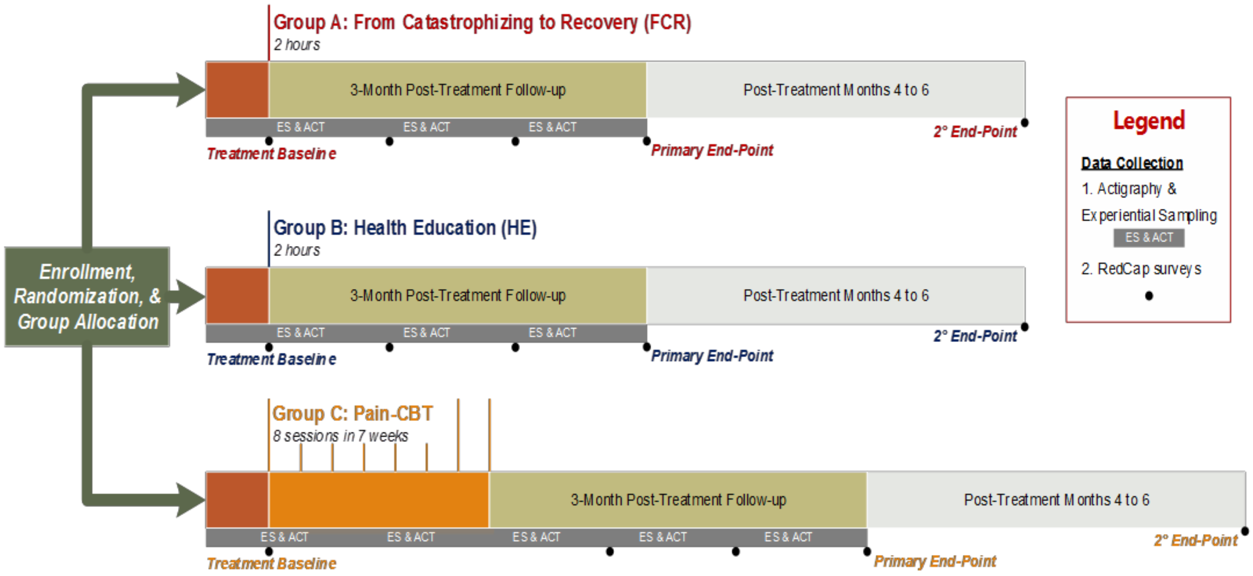

Group A. "**From Catastrophizing to Recovery (FCR)":** A single-session approximately 2-hr group intervention to treat PC.
Format of intervention: Therapist-delivered PowerPoint presentation with experiential exercises. Experienced senior level psychologist and two (2) additional doctoral level psychologists will conduct the FCR sessions. Respectively, instructors include Drs. Valerie Jackson, Kristen Slater, and Maisa Ziadni.
Content of intervention: See Preliminary Study 1 (2014, *J Pain Res*). FCR Participants have the **'FCR** Relaxation Resource' app (guided relaxation audiofile) loaded on their apple or android electronic device. Participants will be given an electronic tablet for the duration of the study if they do no own an appropriate device. Materials for FCR can be found in Appendix 6 and Appendix 7.

Group B. Health Education (HE) Intervention: We aim to control for the non-specific effects of (1) receiving a 'treatment', (2) participating in a research study, and (3) providing daily ratings for PC and pain. The 2-hour HE group will match FCR on 4 important factors: duration, structure, format and site [46].
Format of intervention: The HE class will be expert-led by doctoral level health educator, Kate Lorig, DrPH, and will involve a PowerPoint presentation and handouts. Dr. Lorig and Dr. Darnall have identified, trained and arranged for two (2) additional providers: Dennis Keane and Shannon Fish. The selected instructors have substantial health education experience.
Content of intervention: HE content will include basic information such as nutrition and interacting with the healthcare system; these relatively 'inert' topics are unlikely to impart any specific effects typically found with a true psychobehavioral intervention [46]. Materials for the HE class can be found in Appendix 10.

Group C. 8-week Manualized Pain-CBT Group Intervention: We will test non-inferiority of FCR compared to the current gold standard group treatment for PC-- manualized 8-session pain-CBT.
Format of intervention: Group CBT will be delivered by three (3) PhD-level psychotherapists under the supervision of Beth Darnall, PhD, who has 10+ years experience in the delivery of CBT for pain. The group is run weekly for 8 consecutive classes. Each class is 2 hours with a midpoint break (16 hours total intervention time). Two group cohorts may run concurrently. Groups may be offered at two different times of day to optimize enrollment (e.g., 10am and 4pm). Instructors include Drs. Heather King, Uta Maeda, and Sue Gritzner.
Content of intervention: The protocol and materials, including the therapist manual [47] and patient workbook [48], were developed with funding from NIH/NCCAM R01 AT006226 (Dan Cherkin, PI), and include content from a pain-CBT program developed by Drs. Ehde, Dillworth, and Turner [49]. This specific manualized CBT treatment is also being used in the CBT arm of our NCCIH P01 treatment outcome study for CLBP with Drs. Darnall and King as the CBT therapists), thereby assuring proficiency with the content and delivery of the intervention. We will use treatment fidelity checklists developed by Dr. Cherkin's group (see Appendix 5). The intervention content is included in full in Appendix 3. Participants receive a workbook with homework (see Appendix 4) and a copy of *The Pain Survival Guide: How to reclaim your life* © 2005 by Turk & Winter [51] for optional reading.
Treatment Completion: The threshold for pain-CBT completion is attendance at 5 of the 8 classes. Participants missing more than three (3) classes in total will be considered non-completers. In addition, each missed session will be recorded as a minor protocol deviation.

Treatment Fidelity: A member of the research staff will be trained on the CBT manualized protocol and will assess treatment fidelity using session data and the fidelity checklist (see Appendix 5). He/she will analyze and rate a random sample of 20% of the recorded sessions for fidelity to the manualized CBT protocol. Drs. Darnall or Mackey will review the ratings to confirm fidelity. The research coordinator will

serve as fidelity rater for the FCR class. In structure and format FCR is optimized for treatment fidelity because it has standardized content (PowerPoint presentation; See Appendix 6), and standardized handouts and materials (see Appendix 7). The research coordinator will directly observe the first 3 FCR classes and up to 3 FCR classes at random to ensure treatment fidelity. As an additional measure, we will consider audiotaping the class to maintain blinding of the PI. Dr. Darnall is review audio recordings as needed. In addition, the research coordinator will complete the fidelity sheets for the HE class (see Appendix 11). Fidelity is built into the HE class as well with the use of PowerPoint that makes the curriculum very structured and difficult to deviate from.

Cohorts: Randomization will take place at the individual level to one of the three interventions. Recruitment and screening operations will target having class cohorts of 7-12 participant (minimum of 4 participants, maximum of 20 participants per cohort) for all interventions. Cohort effects are likely to be minimal for FCR and HE owing to their single-session format and relatively minimal participant interaction. For pain-CBT, we will control for any cohort effects in the analyses. This study may leverage CBT classes scheduled for the P01 CAM Center study when space is available.

4. SELECTION AND ENROLLMENT OF PARTICIPANTS

The project will enroll 231 adults (ages 18-70) who meet criteria for axial low back pain without radicular symptoms. Eligibility will be assessed by the research staff. Participants will be randomized to one of the 3 treatment conditions using a random number generator.

To account for attrition we will enroll 231 participants and randomize each individual to one of 3 treatment conditions.

4.1 Inclusion Criteria

Participants must meet all of the following inclusion criteria in order to be enrolled in the study.

- Axial low back pain without radicular symptoms[1]
- Pain duration ≥ 6 months (per recent NIH Task Force on Research Standards for Chronic Low Back Pain [44] (Dr. Mackey was a committee member and guideline coauthor) based on participant self-report
- Average pain intensity ≥4/10 for the past month at screening visit
- English fluency
- Males and females 18-70 years of age
- PCS score ≥20[2] [14]

4.2 Exclusion Criteria

[1] Radicular symptoms will be assessed with an initial set of questions on the online screening form (OSF). If participant indicates yes to those questions, a research team member will follow-up over the phone and if appropriate, the participant will be scheduled for a screening visit. At the visit, a trained research team member will conduct a brief physical assessment of radicular symptoms/radiculopathy for those indicating symptoms (Brief Physical Exam Assessment, Appendix 36).

[2] PCS score ≥20 will be assessed from the online screening form (OSF); scores will be valid for eligibility for 15 days from the date of OSF completion. If the date of OSF completion has passed the 15-day window, the PCS score will be re-administered on the follow-up prior to scheduling a screening visit and/or during the screening visit. For consistency, irrespective of when the OSF PCS was taken, all participants will complete the PCS at screening as deemed by the Schedule of Events (Section 6).

All candidates meeting any of the exclusion criteria at screening will be excluded from the study.

- Gross cognitive impairment
- Active suicidal ideation or severe depression
- Previous attendance in the active treatment groups (any FCR classes ever taken or CBT in the past 3 years)
- Participating in any interventional research study or completed participation in the last 2 months; enrollment in an observational study is acceptable
- Current substance abuse
- Clear likelihood to disrupt fellow class participants (e.g., personality disorder) at the discretion of the study team
- Any radicular symptoms
- Ongoing legal or disability claim, Worker's Comp (permanent and stationary disability not exclusionary)
- Currently pregnant or planning to become pregnant
- Average pain intensity <4/10 for the past month at screening visit
- Disorders indicated by the MINI 7.0 self-report questionnaire will be characterized and participants may be excluded at the discretion of the researcher (e.g. social anxiety disorder would inhibit a person’s ability to fully participate in group treatment)

4.3 **Study Enrollment Procedures**

4.3.1 Recruitment

Craigslist ads: Weekly and monthly Craigslist ads may be placed to advertise for the study. These ads will include basic details about the study and contact information for the study recruiter.

Media ads: The study will optimize use of social media marketing.

Flyers and brochures in the community: We may distribute study flyers and brochures throughout the community at locations such as cafes, restaurants, yoga studios, gyms, YMCAs, libraries, et cetera.

Community clinics: We may distribute study flyers at local clinics and meet with primary care physicians to educate them about the study and provide them with recruitment materials.

Stanford Pain Clinic: All patients at the Stanford Pain Clinic receive an OK to Contact for Research form. If they grant permission, the study team can contact individuals with chronic low back pain to assess their interest and eligibility.

Clinicaltrials.gov posting: Interested patients may contact the study team at the contact information provided on the ClinicalTrials.gov and the Stanford Clinical Trials websites.

SNAPL Database: Individuals in the database with an ok-to-contact form in place will be contacted about the study, including those found ineligible for the P01 CAM Center for Back Pain study if they appear to meet criteria for this study.

<u>Eligibility Screening and Screening Logs</u>

Once potentially eligible individuals are identified, they will be screened over the phone or will fill out an online screening form to determine basic eligibility. A record of all screens will be kept in the Screening Log including screen result, reason for ineligibility, and reason of lack of interest by the individual.

Eligible and Interested Participants

Eligible and interested individuals will be invited to our site for a Screening Visit. At this visit, they will go through Step 1 of the Consenting Procedure detailed in the section 6.2.1. If eligible, they will go through Step 2 of the consenting process and will be enrolled as participants and randomized after consent. Participants will also receive an electronic tablet if they do not have an appropriate electronic handheld device to complete their daily questions on. Participant compliance will be monitored remotely during the baseline period, and they will also be issued reminders. During this period if participants indicate that they have developed an illness, have travel plans, or may not be able to complete baseline and treatment visits on the schedule discussed, their participation will be deferred and they will be invited to participate again after resolution of the issue(s). At the end of the deferral period (up to 6 months), their updated baseline data may be collected. To establish an accurate pre-treatment baseline for participants, the period between baseline data and start of treatment should be within one month.

Individuals not Interested or Ineligible

Individuals who are not interested or ineligible will be asked if they want their information to be included in the SNAPL Database so that they may be contacted for future studies that are of interest to them.

Database Registration

The Research Staff will register all consented participant into the SNAPL Central Database with their unique study ID number and demographic information. Each participant will have two registration records under the same study ID number; one for Screening Consent and the other for Study Consent with the date of each consent.

Recruitment Contingency Plan

We have a recruitment plan in place to ensure steady enrollment. This includes recurring advertisements on Craigslist, local newspapers, direct mailers, and a presence on the social media. In addition, we will plan to run a series of radio ads if enrollment lags. The study recruiter will be contacting community clinics, primary care physicians, free clinics, yoga studios, gymnasiums, and other such facilities to give talks on the Center for community outreach and to educate and build relationships with practitioners who can refer participants to us.

4.3.2 Randomization

Participants (N=231) will be randomized to one of the 3 treatment conditions. An automatic program in REDCap will randomly assign a participant to a group when enrolled. The program will - ensure equal numbers in all three treatment groups at the end of data collection. The use of REDCap will ensure blinded randomization.

5. STUDY INTERVENTIONS

5.1 Interventions, Administration, and Duration

Participants will be randomized into one of three treatment arms: FCR, 8-week CBT, and Health Education. Participants in the CBT group will attend weekly sessions for 8 weeks. Each session will last 2 hours. Participants in the FCR and Health Education classes will attend a single 2-hour session. All treatment sessions will occur at Stanford Pain Clinic, 450 Broadway Street, Redwood City, CA or at Stanford Systems Neuroscience and Pain Lab, 1070 Arastradero Rd., Palo Alto, CA.

5.2 Handling of Study Interventions

A member of the research staff will be trained on the CBT manualized protocol and will assess treatment fidelity using session recordings and the fidelity checklist (see Appendix 5). He/she will analyze and rate a random sample of 20% of the recorded sessions for fidelity to the manualized CBT protocol. Drs. Darnall or Mackey will review all rating forms to confirm fidelity. The research coordinator will serve as fidelity rater for the FCR class. The research coordinator will complete the fidelity sheets for the HE class (see Appendix 11). In structure and format FCR and the HE class are optimized for treatment fidelity because they have standardized content (PowerPoint presentation; Appendix 6, Appendix 10), and standardized handouts and materials. The research coordinator will observe the first 3 FCR classes and another 3 FCR classes at random to ensure treatment fidelity. In addition, class instructors will also be audiotaped for Dr. Darnall to review as needed. This will enable her to remain blinded and ensure FCR class treatment fidelity.

The general approach for delivering the intervention is a classroom setting with instructors following a strict, pre-defined protocol for each session. The details for each intervention are given below in section 6.2.4.

5.3 Concomitant Interventions

5.3.1 Allowed Interventions

Participants may continue their current medication/intervention regimen throughout the study if the regimen does not include any intervention listed in “Prohibited interventions” below. Medication/intervention use will be recorded through the questionnaires given to participants in the study.

5.3.2 Required Interventions

N/A

5.3.3 Prohibited Interventions

- N/A

5.4 Adherence Assessment

5.4.1 Protocol Adherence and Treatment Fidelity

We will use a treatment fidelity scale that has been developed for CBT. A member of the research staff will be trained on the manualized protocol and will assess treatment fidelity using session data and the fidelity checklist (see Appendix 5). He/she will analyze and rate a random sample of 20% of the recorded sessions for fidelity to the manualized CBT protocol. Drs. Darnall or Mackey will review the ratings to confirm fidelity. The research coordinator will serve as fidelity rater for the FCR class. In structure and format FCR is optimized for treatment fidelity because it has standardized content (PowerPoint presentation; Appendix 6), and standardized handouts and materials (Appendix 7). The study coordinator will observe the first 3 FCR classes and up to 3 FCR classes at random to ensure treatment fidelity. FCR classes will also be audio recorded and reviewed by Dr. Darnall as needed. The research coordinator will complete the fidelity sheets for the HE class (see Appendix 11). Fidelity is built into the HE class as well with the use of PowerPoint that makes the curriculum very structured and difficult to deviate from.

We provide each instructor with the criteria that will be used to make the ratings of treatment fidelity.

5.4.2 Daily Questions

At specified time points during study involvement, participants will complete a daily questionnaire. This typically takes less than 3 minutes to complete each day. Reminders are sent daily through email or text message and responses are entered online through REDCap.

Daily questions will be captured in two-week increments at each of the following time-point:Pre- treatment, mid-treatment (for 8-week CBT), post-treatment 0, 1, 2, and 3 months post-treatment.

Daily skills use (3 questions) will only be asked for FCR and CBT participants post-treatment as the skills use questions will not be relevant to HE participants.

5.4.3 Treatment Attendance

Participant attendance at each treatment session will be recorded and the research team will follow up with participants who miss sessions.

5.4.4 Measures to Promote Adherence and Participant Retention

Ensuring participant understanding of study expectations:

- Knowledgeable and receptive staff: Staff education plan above ensures competent description of the study process and expectations. Staff are selected for the ability to communicate study expectations and process clearly and engage the participant in the process.
- Providing a Welcome Packet with study information and easily found contact information for study staff.

Participant Retention Measures

- Developing a personal relationship with the participant.
- Friendly staff: Staff will also be selected for their interpersonal skills and ability to engage the participant in a friendly conversation.
- Providing a thank you note to participants for participating in the study.

FCR and HE groups benefit from having low attrition because they are single-session treatments. However, the CBT treatment group has a high risk for attrition because it involves 8 group treatment sessions. The study coordinator will call participants following any absences, will inquire about their health, and encourage them to attend the next class. We will monitor attrition in the CBT group and if in Year 3 could implement an adaptive randomization scheme if inequality in treatment group size threatened our statistical power / ability to answer our scientific questions. To minimize attrition in all 3 groups during the follow-up phase, the study coordinator will maintain close contact with participants to ensure data collection, identify any problems, and confer with the PIs to develop and implement solutions. Finally, our metered payment scheme rewards completion of the study.

Participant Attrition: Our plan for over enrollment will allow for realistic study attrition while preserving desired group census and adequate power. Participants who drop out of treatment will be compensated to provide ongoing data. If willing, non-completers will be requested to complete all relevant data/questionnaires. We will record participant reasons for study attrition for *all* conditions,

and will analyze all available data to characterize non-completers.

5.4.5 Specific Triggers and Stopping Rules

FCR – If a participant does not show, at the last minute can no longer attend, or arrives >15 minutes after the start of the class, the participant will be reassigned to the next session of FCR.

HE – If a participant does not show, at the last minute can no longer attend, or arrives >15 minutes after the start of the class, the participant will be reassigned to the next session of HE.

CBT – If participants in the CBT arm of the study miss more than 3 visits, their participation will be considered incomplete. They may participate in the remaining sessions if they wish. We will perform intention to treat analyses and will examine number of sessions completed as a potential moderator variable.

Missing more than 3 visits for CBT will likely yield less than optimal treatment outcome results for making required inferences. We will still be analyzing all available data for non-completers to further our understanding of chronic low back pain. If willing, all non-completers will be requested to complete all relevant data/questionnaires.

Please note: Each missed session will be recorded as a minor protocol deviation.

6. STUDY PROCEDURES

6.1 **Schedule of Evaluations** -- please see table below.

Schedule of Events

Measurement Domain/ Name of Measure	Brief Measure Description	Online Screening Form (OSF)[3]	Screening	Baseline Period	Pre-treatment	Mid-treatment	Post-tx Month 0	Post-tx Months 1, 2 3, 5 & 6
Demographics	Age, gender, race, ethnicity, handedness, education level, household income, employment.		x					
Medical History and Medications	Height, weight, smoking, back pain etiology, pain duration, pain intensity, other pain conditions, pain treatments, psychological conditions, medications.		x					
Mini International Neuropsychiatric Interview (MINI) [52]	A MINI screen will be administered for two reasons: (1) for exclusionary criteria assessment for suicidality and current substance abuse; (2) to characterize other psychiatric disorders. Positive items will be followed by administering relevant modules of the Beck Depression Inventory-II (BDI-II) and Structured Clinical Interview for DSM (SCID) by a trained member of the study team.		x					
Beck Depression Inventory-II (BDI-II)	If an individual meets the criteria for depression on the MINI Screening, then they will be assessed further with the BDI-II to establish symptomatic quantification and severity.		As needed					
Structured Clinical Interview for Diagnosis and Statistical Manual of Mental Disorders, Fifth Edition (SCID-5)	If the individual scores > 26 on the BDI-II (above mean for moderate depression), an onsite psychologist will be notified to conduct the depression module for the SCID-5-CV.		As needed					
Radicular Assessment (Brief Physical Exam)	Nine (9)-question physical assessment for radicular symptoms and/or radiculopathy. Assessment will be done on participants whc indicate pain radiating down leg(s).		As needed					
Chronic Pain Acceptance Questionnaire (CPAQ-8)	Eight (8) question version CPAQ has been designed to measure acceptance of pain. The acceptance of chronic pain is thought to reduce unsuccessful attempts to avoid or control pain and thus focus on engaging in valued activities and pursuing meaningful goals.				x	x	x	x
West Haven-Yale Multidimensional Pain Inventory (WHY-MPI)	WHY-MPI is designed to provide a brief, psychometric assessment of important components of the chronic pain experience. In particular, this study will be looking at how a significant other responds to a participant when they are in pain.				x	x	x	x
Body Map	Interactive map of male/female body to select regions that experience pain.				x	x	x	x
Back Pain Bothersomeness	Single item measure of back pain bothersomeness in the past week from not at all bothersome to extremely bothersome.				x	x	x	x
Satisfaction with Life Scale [77]	A 5-item scale designed to measure global cognitive judgments of one's life satisfaction. Participants indicate how much they agree or disagree with each of the 5 items using a 7-point scale that ranges from 7 strongly agree to 1 strongly disagree.				x	x	x	x

[3] Online Screening Form (OSF): A survey consisting of questions based on eligibility criteria to determine initial eligibility for invitation to an in-person screening visit. This questionnaire consists of questions on age, gender, name, address, source of information, chronic low back pain duration, average level of back pain, radicular symptoms, pregnancy, past pain psychology treatments, PCS, worker's compensation, and concurrent studies, The OSF streamlines study recruitment for the team as all participant take this as a first step to determine eligibility.

Perceived Stress Scale [78]	10 items on 5-point scale from never to very often measuring aspects of perceived stress in the past month. Items were designed to measure how unpredictable, uncontrollable, and overloaded respondents perceive their life to be.			x	x	x	x

**Measurement Domain/ Name of Measure**	**Brief Measure Description**	**Screening /OSF**	**Baseline Period**	**Pre-treatment**	**Mid-treatment**	**Post-tx Month 0**	**Post-tx Months 1, 2 3, 5 & 6**
Childhood Trauma Questionnaire [79]	The self-report measure includes 28 items that measure 5 types of maltreatment – emotional, physical, and sexual abuse, and emotional and physical neglect. Items are measured on a 5-point Likert scale with responses ranging from never true to often true.			x			
Positive and Negative Affect Schedule [80]	10 positive affect descriptors and 10 negative affect descriptors are measured for the resent moment on a 5-point scale ranging from very slightly or not at all to extremely.			x	x	x	x
Patient's Global Impression of Change [81]	One item measure of status change since start of treatment and one item measure of side effects.					x	x
Working Alliance Inventory [82]	12 items on 7-point scale from never to always measuring how the participant feels about the treatment group instructor.				x	x	
Treatment expectancies [53]	Stanford Expectations of Treatment Scale, a 6-item tool our group developed and validated at SNAPL, will be used to assess participant expectations of treatment.			x			
Mid Credibility Expectancy Questionnaire [83]	4-items assessing patient impressions of the treatment midway through treatment participation.				x		
Post Credibility Expectancy Questionnaire [83]	4-items assessing patient impressions of the treatment post-treatment participation.					x	
Tx satisfaction, utility, and knowledge	7 items assess participant satisfaction and perceived utility of treatment on a 7-point rating scale. For the FCR group, 5 items will assess knowledge acquired regarding PC and self-treatment.					x	
Treatment and Lifestyle Changes	Assesses new treatments, major lifestyle changes, major life events (negative and positive), and new injuries at different time points throughout the study.			x	x	x	x
Skills Use	Single item measure assessing frequency of skills use learned in class over the past month from not at all to several times per day.						x
<u>**Primary treatment outcome measures**</u>							
Pain Catastrophizing Scale (PCS) [14]	13-item scale assesses severity of trait pain catastrophizing tendencies on a 5-point scale (0 = "not at all"; 4 = "all the time"); sum scores range from 0-52.The PCS has 3 factors (helplessness, magnification, rumination) and has good psychometrics [14]. OSF PCS will be valid for a 15-day period for eligibility. **The PCS total score at 3 months post-treatment is our primary endpoint (Trait PC).**	x	x	x	x	x	x
NIH PROMIS measures [54]	NIH PROMIS measures have been successfully applied in pain research [55-58] and will be used to assess multiple variables of interest, including Pain Intensity, Pain Interference, Pain Behavior, Physical Function including 4 pre-selected Mobility CAT questions, Depression, Anxiety, Sleep Disturbance, Sleep Interference, Anger, and Fatigue. Short forms will be used to minimize participant burden. Participants also complete PROMIS Global Health at the same time points.			x	x	x	x
Pain Self-Efficacy Questionnaire (PSEQ) [60]	10-item instrument measures self-confidence to manage pain and engage in life activities despite pain [23,26,61-64]. Pain self-efficacy be used as a mediating / moderating / process variable.			x	x	x	x

Measurement Domain/ Name of Measure	Brief Measure Description	Screening /OSF	Baseline Period	Pre-treatment	Mid-treatment (CBT Only)	Post-tx Month 0, 1, 2 3
**Daily Measures / ESM (2 week data waves)[4]**						
Daily PCS	The 5-item Daily PCS will be administered daily.		→		→	0-3 mos. →
Daily pain, mood , and function questions	In a single question for each theme, for the past 24 hours, average back pain, highest level of back pain, pain interference, stress, positive emotions, negative emotions, and satisfaction with life as assessed.		→		→	0-3 mos →
Daily skills use	FCR group will answer 3 questions assess use of cognitive, behavioral and psychophysiological techniques over 24 hours from 0 times to 5+ times. The pain-CBT group will complete these questions daily after the relevant material is covered in class.					0-3 mos (only for CBT & FCR) →
**Objective Data**						
Sleep & Activity	ActiGraph GT9X Link devices will quantify 24 hour sleep and activity variables including: total sleep time and efficiency, energy expenditure, MET rates, steps taken, physical activity intensity.		→			Post-Tx only 0 & 3 mos →
Guided Relaxation (per participant discretion/goal setting; continuous data)	An app will timestamp each time an FCR participant accesses the "FCR Relaxation Resource" on their device, thus providing an objective measure of skills use.					0-3 mos (continuous FCR only) →

6.2 Description of Evaluations

6.2.1 Screening Evaluation

These evaluations occur to determine if the candidate is eligible for the study.

Consenting Procedure

Consent Process: Prior to collecting any in-person screening or research information, designated and trained Clinical Core staff will review the participant's IRB-approved Consent Form with him/her in a private area, answer any questions, witness the participant sign the informed consent and write the date consent was obtained, and verify by signing as the research team member that obtained consent. Each participant will receive a signed and dated copy of their Consent Form documents. The consenting process will be divided into two steps.

> **STEP 1:** The participant will first sign a consent form for the study team to collect basic screening information. After they have signed and dated the Screening Consent Form and are eligible to participate in the study (based on details in the Screening section below), they will go to Step 2 of the consenting process.
>
> **STEP 2:** At this step, participants will sign the Study Consent Form and will learn in detail about the study procedures. Research staff will answer all study-related questions and make sure that the participant fully understands all procedures, tests, and visits for the study.

Storage of Consent Forms: Consent forms will be stored in a binder labeled "PHI and Consent Forms for

[4] Completion of ESM Data is defined as completing at least seven (7) days of daily surveys and actigraphy data for each of the 2-week periods.

FCR RCT" and dated. The binder(s) will be stored in locked cabinets. Each consent form will be labeled in the upper right corner with subject ID number.

Screening

The screening process may last several weeks from the time the participants is screened over the phone/online to the time of enrollment.

During the screening visit, individuals will undergo informed consent for eligibility assessment (Screening Consent) as explained in the Consenting Procedure section above. The eligibility assessment will include the following evaluations:

- Demographics and other questionnaires
- Medical History
- Confirmation of meeting inclusion/exclusion criteria[5]
- The MINI 7.0 Self-Report screening questionnaire (follow-up with BDI-II & SCID-5 as needed)

The research coordinator will conduct the MINI screening with each participant and make the determination of eligibility for the study. Research coordinators are Masters level pain researcher and will be trained extensively on the MINI screening prior to administration. To ensure competency, the coordinator will conduct a screening in front of a clinical psychologist prior to administering one-on-one with study participants.

If a patient is determined to be suicidal or severely depressed, the research coordinator will immediately consult the team psychologists/clinicians. Stanford Systems Neuroscience and Pain Lab (SNAPL) has a manualized protocol on how to handle suicidality and severe depression. The psychologist/MD will assess safety.

Phone/Electronic Screens

Initial eligibility will be assessed via telephone or secure, HIPPA-compliant online screen based on the inclusion/exclusion criteria. Individuals who meet preliminary criteria will be scheduled for their Screening Visit.

Eligibility Determination

The points where a participant may be considered ineligible for the study are as follows:

- Phone/online screen: If participants do not meet the preliminary criteria, they will be informed of their ineligibility
- Screening Visit: At the screening visit, the participant's eligibility will be determined by collecting answers to questionnaires, the medical history and by results from the MINI. If the participant meets exclusion criteria, he/she will be excluded from participating in the study.

Eligible and Interested Participants

Participants who are interested and eligible will do the following:

---

[5] As mentioned above, PCS score ≥20 will be assessed from the online screening form (OSF); scores will be valid for eligibility for 15 days from the date of OSF completion. If the date of OSF completion has passed the 15-day window, the PCS score will be re-administered on the follow-up prior to scheduling a screening visit and/or during the screening visit.

- **Treatment Visit Scheduling:** Interested and eligible and who have signed the Study Consent Form will be scheduled for their treatment.
- **Daily Survey and Actigraphy Instruction:** Participants will be given an actigraphy unit. Daily surveys will be administered by email. IF participants do not own a smart, electronic, handheld device, we will provide them with an electronic device to facilitate responding to daily questions about their pain, mood, and physical functioning; and, if in the FCR condition, the device will facilitate use of the 'Relaxation Resource'. They will be instructed on how to use the electronic device and will be asked to complete the daily reports before going to bed each night. Participant compliance will be monitored remotely during the baseline period, and they will also be issued reminders.
- **Welcome Packet:** Enrolled participants will receive a welcome packet containing copies of their consent forms, their treatment information, a timeline of their participation, and relevant contact numbers.

Participants not interested or ineligible

Participants who are not interested or ineligible will be asked if they want their information to be included in the SNAPL Central Database so that they may be contacted for future studies that are of interest to them.

6.2.2 Enrollment, Baseline, and/or Randomization

Enrollment

If all eligibility criteria are met as stated above, the participant will undergo Step 2 of the Consenting Procedure and mentioned above in the Consenting Procedure section. This is the point of enrollment of the participant into the study. The enrollment date will be recorded in the SNAPL Central Database. The enrollment date will also be recorded on the Screening and Enrollment CRF, which will have a schedule of future visits.

Randomization

Randomization to a specific treatment arm will take place after the study consent is completed at the screening visit. This allows for immediate scheduling of treatment, an essential element that will facilitate participant retention and will minimize unequal wait times.

Baseline

Participants will begin completing two weeks of daily questionnaires and actigraphy 2 weeks prior to treatment start and within a month of treatment start[6]. The 2-week pre-treatment baseline period will **not** be used to determine eligibility. Pre-treatment baseline data will be examined at final analyses only.

6.2.3 Blinding

Participants will not be blinded to the intervention they are randomized to. Treatment providers and study coordinators will not and cannot be blinded to treatment allocation. The study coordinator will be responsible for handling the randomization process through REDCap; however, they are blinded to the randomization scheme. This study coordinator will coordinate delivery of intervention; therefore, will not have access to data, data monitoring, or analysis of data. An alternative research team member will

[6] The end of the baseline period will be within 30 days of treatment start date.

have access to the data and will be responsible for the data monitoring. The PIs and Co-Investigators will remain blinded through the data collection phase.

An independent biostatistician, Nhat Hoang, will generate the randomization sequence based on the protocol prior to the study, will store and forward it in a blinded manner to study coordinators. Mr. Hoang will not be involved in interim or final analyses.

Dr. Ming Kao, our study Co-Investigator and Dr. Lu Tian will be involved with the interim and final analysis and will remain blinded throughout the study.

6.2.4 Treatment Visits

We are optimizing systems such that a new treatment group will aim to launch every 8-10 weeks, regardless of treatment arm. We aim to deliver treatment within 8-10 weeks of enrolling a participant. Preferably, participants will wait no longer than 8 weeks to begin engaging in the study after the 2-week baseline period daily assessment period. All pre-treatment assessments will be captured up to 3 days before starting treatment.

To address the long periods between randomization and intervention statistically, we have worked with our Co-Investigator and biostatistician, Dr. Ming Kao. We are implementing practical elements to minimize differential drop-out rates due to wait time (e.g., we are ensuring equal wait times for treatment, and therefore providing equal access to treatment). Please see Section 9.2 for further details.

CBT: We will be using the same CBT treatment manual as Dan Cherkin, PhD, who is conducting a NCCAM/NCCIH funded RCT of CBT versus MBSR for participants with chronic lower back pain and as the P01 Stanford CAM Center for Back Pain Study.

Below is an outline for the 8 classes included in the CBT intervention.

Session	Content	Relaxation	Activit y [illegible]	Coping Skill	Home Activities
**1**	Welcome & Intros (incl. group rules, logistics, etc.); CBT rationale; Pain physio; Relaxation rationale; Importance of home practice	Diaphragmatic breathing (this is practiced at each session)	Rationale for setting physical activity goals		Practice diaphragmatic breathing; Set & record physical activity goal
**2**	Goal setting, activation, & pacing (SMART, rest-activity cycle, etc.); Red flags; Coping with flare-ups & creating a flare-up plan	7-muscle group PMR	Continue w/ physical activity goal; Choose pleasant activity goal		Practice diaphragmatic breathing & 7-muscle group PMR; Work on physical & pleasant activity goals; Practice pacing; Use flare-up plan, if needed
**3**	Role of thoughts & feelings in pain; Into to CBT & terms; Intro to 3-column thought record	4-muscle group PMR; Fitting diaphragmatic breathing into your daily life	Continue modifying & working on physical & pleasant activity goals		Practice breathing & 4- muscle group PMR; Work on activity goals; Continue to be mindful of pacing; Complete at least 1 thought record; Use flare-up plan, if needed

**4**	Evaluating & generating alternate thoughts; Intro to evidence gathering; Intro to 4-column thought record	4-muscle group PMR, no tension	Continue modifying & working on physical & pleasant activity goals	Coping thoughts	Practice breathing & 4- muscle group PMR, no tension; Work on activity goals; Continue to be mindful of pacing; Complete at least 1 thought record; Come up with at least 3 coping thoughts; Use flare-up plan, if needed

Session	Content	Relaxation	Activity Goals	Coping Skill	Home Activities
**5**	More on evidence gathering & alternate thoughts (more detail); Working with thoughts review;	Body Scan	Continue modifying & working on physical & pleasant activity goals	Distraction	Practice breathing & body scan; Work on activity goals; Continue to be mindful of pacing; Complete at least 1 thought record; Practice distraction; Use flare-up plan, if needed
**6**	Thought records review	Walking body scan	Continue modifying & working on physical & pleasant activity goals	Thought stopping	Practice breathing & walking body scan; Work on activity goals; Continue to be mindful of pacing; Complete at least 1 thought record; Practice thought stopping; Use flare-up plan, if needed
**7**	Review of skills; Trouble-shooting re: thought records; Pain & mood; Pain core beliefs		Continue modifying & working on physical & pleasant activity goals	Sleep Tips	Practice breathing & mini-relaxation; Work on activity goals; Continue to be mindful of pacing; Complete at least 1 thought record; Complete at least 1 Pain Belief record (if applicable); Practice any or all of Bx skills; Practice sleep tips; Use flare-up plan, if needed
**8**	Review of skills; “Signs” not using skills: Creating a plan for maintaining gains & dealing with setbacks; Termination & wrap-up	Guided imagery	Continue modifying & working on physical & pleasant activity goals		Practice guided imagery or any of the relaxations; Work on activity goals; Continue to be mindful of pacing; Complete thought records as needed; Practice any or all of Bx skills; Practice sleep tips; Use flare-up plan, if needed; Add to or update relapse plan as needed

FCR: FCR will be delivered via a PowerPoint presentation to groups of participants in a single session lasting approximately 2.5 hours.

Content: FCR has two main components: didactics and skills acquisition. Didactic content includes mind–body science as it relates to pain and PC. Participants learn how to identify catastrophizing in the moment, and how to self-treat it. During the class, participants acquire skills and develop a plan to apply the learned skills to decrease physiological hyperarousal – diaphragmatic breathing and progressive muscle relaxation – within the context of PC. Participants also acquire skills that improve the regulation of cognition and emotion, including PC reframing and thought restructuring, and develop a plan for implementing these skills in daily life. During the class participants identify their typical PC thoughts and practice writing out their reframes. Finally, participants develop a plan to use behaviors that modulate attention and counteract helplessness. During the class, participants create personalized lists of self-soothing behaviors used to stop PC; lists are customized to various settings. Participants self-tailor the information relayed during the class by developing their own comprehensive self-treatment plan to stop and prevent catastrophizing. Participants leave the class with the following tangibles: 1) their self-written, self-crafted, personalized PC cessation plan; 2) a 20-minute relaxation response audio CD; and 3) a printed copy of the FCR content to access as needed in their PC cessation plan.

HE Class: The HE class will be delivered via a PowerPoint presentation to groups of participants in a single session lasting approximately 2 hours.

Content: The HE class will give an overview of back pain, including common sources and red flag symptoms. It will also cover topics including managing a pain flare-up, working with healthcare professionals, evaluating treatments and making informed treatment decisions, and achieving and maintaining a healthy weight through good nutrition and exercise.

Online Follow-Up: Participants will complete online assessments 0, 1, 2, 3, and 6 months (optional) post-treatment in addition to at 5-month time point for FCR and HE participants. CBT will also complete a mid-treatment questionnaire battery. The study staff will also make phone calls to remind participants to complete the online follow-up questionnaires.

6.2.5 Assessments

Subjective Data

We will collect subjective data using daily sampling methods (ESM) and questionnaires administered at enrollment, class baseline, mid-treatment (CBT only), post-treatment (end of class), and post-treatment at months 1, 2, 3 and 6 (optional).

Experiential Sampling Methods (ESM): Daily questionnaires will be administered through email link disbursed to participants daily during the designated 14-day discrete data waves. Dr. Kao (Co-I) has specific expertise with implementation and optimization of ESM and actigraphy in the research context, and relevant analytic expertise [41]. ESM advantages include: (1) it provides a strong longitudinal database to assess changes in daily pain and psychological symptoms over time; (2) data are regularly uploaded to the database, thus allowing for compliance monitoring; (3) prevents recall bias associated with retrospective ratings; (4) allows us to characterize mean Daily PC and its impact on pain, Trait PC and other variables (Aim 2).

If participants do not have a smart, handheld electronic device, they will receive a tablet for the purpose of ESM--inputting daily ratings for pain, PC, and skills use. However, only FCR participants will have the "FCR Relaxation Resource" app loaded on their devices. The devices will (1) be locked to prevent use that is unrelated to the study (e.g. email or internet searches); (2) programmed with automatic

reminders that alert participants to input their data at the end of each day; (3) have data upload automatically to the secure study server for participants who have home WiFi. Data will store locally on the device for participants without WiFi at home; they will be instructed to pass by or visit a WiFi hotspot (e.g., a Starbucks) with the device weekly for automatic data uploading to the secure study server. The study coordinator will conduct data checks every few days and will follow compliance and participant retention optimization plans.

Objective Data

Actigraphy: We will use the accelerometry / actigraphy device, ActiGraph GT9X Link, to objectively quantify sleep and activity variables. Actigraphy has been shown to provide an objective assessment of functional changes in multiple chronic pain disorders, including CLBP [66-69]. Actigraphy provides a measurement of function that is long-term, physical in origin, and free-living in context [69]. The small, lightweight devices are worn on the non-dominant wrist and provide objective data for 24-hour sleep/wake measurement (e.g, total sleep time, sleep efficiency) and activity level (energy expenditure, MET rates, steps taken, physical activity intensity). *Sleep and activity were targeted for objective measurement based on our pilot findings (See Preliminary Study 2) showing that FCR was associated with longitudinal improvements in these domains* [70]. The devices store 120 days' of data thus precluding need to visit the study site for data downloads.

Relaxation Resource App for the FCR group: FCR participants will have on their handheld, electronic device an already-developed 'Relaxation Resource' app that they will use to access a relaxation audiofile Appendix 8. The app will be refined with a time-stamp feature that will provide an objective measure of use. This "compliance" tracking--combined with 3 daily ESM questions that assess skills use--will closely track participant behavior and thus will inform our understanding of the *mechanisms* of treatment and change, as was recommended by authors of a review on mechanisms of catastrophizing and CBT [42].

6.2.6 Study Completion

- **Project Stop Point CRF:** Research staff will complete second half of project stop point CRF after participant completes their last online follow-up. At this point, it will be indicated on the CRF that all follow-up visits are complete and the participant's record will be closed.
- **Participant payment:** Participants will receive $25 for the in-person screening visit. For the study, up to $425 total compensation is possible for participating in the study, completing 3-month ESM data (defined as completing - up to 7 missing daily data entries for each designated 2-week ESM data), and completion of the post-treatment follow-ups for months 0, 1, 2, 3, 5, and 6. Please see the compensation scheme in Appendix for details. We had success with similar payment plans in other studies [8]. Travel payment: $10 will be paid for any visit required for data downloads.

Note: All post-treatment surveys must be completed within 3 weeks following each post-treatment time point. We have multiple systems in place to ensure data is collected within days of survey deployment. Our survey system will close and locked 3 weeks following survey. All data will be time-stamped in the event NCCIH requires a smaller window.

7. SAFETY ASSESSMENTS

There are no adverse events anticipated from the CBT, FCR, or HE.

7.1 Specification of Safety Parameters

Not applicable

7.2 Methods and Timing for Assessing, Recording, and Analyzing Safety Parameters

Not applicable

7.3 Adverse Events and Serious Adverse Events

An **adverse event (AE)** is generally defined as any unfavorable and unintended diagnosis, symptom, sign, syndrome or disease which either occurs during the study, having been absent at baseline, or if present at baseline, appears to worsen. Adverse events are to be recording regardless of their relationship to the study intervention.

A **serious adverse event (SAE)** is generally defined as any untoward medical occurrence that results in death, is life threatening, requires inpatient hospitalization or prolongation of existing hospitalization, results in persistent or significant disability/incapacity, or is a congenital anomaly.

There are no SAEs anticipated for this research study.

7.4 Reporting Procedures

Adverse Events will be reported to NCCIH, DSMB, and Stanford IRB annually. Serious Adverse Events that are determined to be related to the study will be reported to the IRB by filing a report on the Stanford IRB website. A copy of this report will be sent to the NCCIH officer. Unexpected fatal or life-threatening Adverse Events related to the intervention will be reported to the NCCIH Program Officer within 7 days. Other serious, unexpected, and related Adverse Events will be reported to the NCCIH Program Official within 15 days and to the Stanford IRB within 10 business days. Anticipated or unrelated Serious Adverse Events will be handled in a less urgent manner but will be reported to the Independent Monitor(s), Stanford IRB, and NCCIH in accordance with their requirements. In the annual Adverse Event summary, the Independent Monitor(s) Report will state that they have reviewed all Adverse Event reports.

7.5 Follow-up for Adverse Events

After Adverse Events are discussed by the Investigator and participant and a decision is made regarding continuation of participation, the study coordinator will conduct a 1-month follow-up phone call to the participant. This phone call will also be recorded in the Adverse Event CRF.

7.6 Safety Monitoring

DATA SAFETY AND MONITORING BOARD (DSMB)

The DSMB has the following members:

Dr. Bruce Arnow, PhD (Clinical psychiatry expertise)

Dr. Leanne Williams, PhD (Clinical RCT and data integrity expertise)

Dr. Tina M. Hernandez-Boussard, PhD, MPH, MS (Patient safety and biostatistics expertise)

The DSMB will convene once a year and as per needed basis. Safety will be reviewed and participant

cases will be reported to the members of the DSMB and relevant safety decisions will be made. The sessions will be open to the Project Leaders and PIs. DSMB reports will be sent to NCCIH and Stanford IRB within 10 business days of the meeting.

8. INTERVENTION DISCONTINUATION

Participants will be free to withdraw from the study if they no longer wish to participate. If participants miss more than three sessions of the CBT arm, their participation will be considered incomplete. However, they will not be withdrawn from the study and will be given the opportunity to resume classes. Since this study is not monitoring any lab values and does not involve drugs, there are no such values that would warrant intervention discontinuation. Participants will be withdrawn from the study by the Investigator if it is determined that the interventions are causing the negative mood and it cannot be resolved.

9. STATISTICAL CONSIDERATIONS

9.1 General Design Issues

Aim 1: Implement a comparative efficacy trial of "From Catastrophizing to Recovery" (FCR)

For Aim 1, our *overall hypotheses* are that, with regard to reducing PC, the 2-hour FCR class is: (1) superior to the 2-hour Health Education (HE) class and (2) non-inferior to an 8-session pain-CBT class. We will also assess assay sensitivity in the non-inferiority trial by testing the hypothesis that pain-CBT is superior to the HE class.

Aim 2: Characterize the mechanistic influence of Daily PC on future pain, function, and Trait PC

We will use multi-level modeling to characterize treatment effects on Daily PC fluctuations, and the impact of Daily PC changes on Trait PC, longitudinal PROMIS measures, and actigraphy (activity and sleep).

9.2 Sample Size and Randomization

<u>Participants</u>

The project will enroll 231 adults (ages 18-70) who meet criteria for axial low back pain without radicular symptoms. Eligibility will be assessed by the research staff. Participants will be randomized to one of the 3 treatment conditions.

<u>Treatment Assignment Procedures</u>

Participants (N=231) will be randomized to one of the 3 treatment conditions. An automatic program in REDCap will randomly assign a participant to a group when enrolled. The program will ensure equal numbers in all three treatment groups at the end of data collection. The use of REDCap will ensure blinded randomization.

To address the long periods between randomization and intervention statistically, we have worked with our Co-Investigator and biostatistician, Dr. Ming Kao. We are implementing practical elements to minimize differential drop-out rates due to wait time (e.g., we are ensuring equal wait times for treatment, and therefore providing equal access to treatment). We will over enroll to account for possible differential drop-out rates. We will also closely monitor drop-out rates during the study. In short, efforts will be made

to minimize differential drop out and we will assess and account for any differential drop out (should it occur) in the analyses.

<u>Selection Criteria</u>

These selection criteria derive from our analysis of the tradeoff between using highly restrictive inclusion criteria in order to have a "pure" sample, and using few inclusion criteria to increase generalizability of results to the community. Participants must meet all of the following inclusion criteria in order to be enrolled in the study:

- Axial low back pain without radicular symptoms[7]
- Pain duration ≥ 6 months (per recent NIH Task Force on Research Standards for Chronic Low Back Pain [44] (Dr. Mackey was a committee member and guideline coauthor) based on participant self-report
- Average pain intensity ≥4/10 for the past month at screening visit
- English fluency
- Males and females 18-70 years of age
- PCS score ≥ 20 [14][8]

All candidates meeting any of the exclusion criteria below at screening will be excluded from the study.

- Gross cognitive impairment
- Active suicidal ideation or severe depression
- Previous attendance in the active treatment groups (any FCR classes ever taken or CBT in the past 3 years)
- Participating in any interventional research study or completed participation in the last 2 months; enrollment in an observational study is acceptable Current substance abuse
- Clear likelihood to disrupt fellow class participants (e.g., personality disorder) at the discretion of the study team
- Any radicular symptoms
- Ongoing legal or disability claim, Worker's Comp (permanent and stationary disability not exclusionary)
- Currently pregnant or planning to become pregnant
- Average pain intensity <4/10 for the past month at screening visit
- Disorders indicated by the MINI 7.0 self-report questionnaire will be characterized and participants may be excluded at the discretion of the researcher (e.g. social anxiety disorder would inhibit a person's ability to fully participate in group treatment)

---

[7] As mentioned above, radicular symptoms will be assessed with an initial set of questions on the online screening form (OSF). If participant indicates yes to those questions, a research team member will follow-up over the phone and if appropriate, the participant will be scheduled for a screening visit. At the visit, a trained research team member will conduct a brief physical assessment of radicular symptoms/radiculopathy for those indicating symptoms (Brief Physical Exam Assessment, Appendix 36).

[8] Also as mentioned above, PCS score ≥20 will be assessed from the online screening form (OSF); scores will be valid for eligibility for 15 days from the date of OSF completion. If the date of OSF completion has passed the 15-day window, the PCS score will be re-administered on the follow-up prior to scheduling a screening visit and/or during the screening visit.

9.3 **Definition of Populations**

ITT (Intent to treat) refers to all participants who have completed the screening visit and have been randomized to 1 of 3 arms.

Per protocol refers to participants who have completed treatment and the post-treatment assessment.

9.4 **Interim Analyses and Stopping Rules**

The treatments in this study are not associated with risks.

This study will be stopped prior to its completion if: (1) the intervention is associated with adverse effects that call into question the safety of the intervention; (2) difficulty in study recruitment or retention will significantly impact the ability to evaluate the study endpoints; (3) any new information becomes available during the trial that necessitates stopping the trial; or (4) other situations occur that might warrant stopping the trial.

We do not anticipate any adverse effects from this intervention.

9.5 **Outcomes**

Our design is a randomized 3-arm study with test treatment, active control, and placebo arms. Our goals are to provide scientific evidence to demonstrate the efficacy of FCR, and also provide a comparison of said efficacy against the standard of care – group pain-CBT. Treatment allocation is randomized to minimize confounder effects. Statisticians performing analyses will be blinded.

9.5.1 Primary Outcome

Aim 1: Implement a comparative efficacy trial of "From Catastrophizing to Recovery" (FCR)

For Aim 1, our *overall hypotheses* are that, with regard to reducing PC, the 2-hour FCR class is: (1) superior to the 2-hour Health Education (HE) class and (2) non-inferior to an 8-session pain-CBT class. We will also assess assay sensitivity in the non-inferiority trial by testing the hypothesis that pain-CBT is superior to the HE class.

<u>Primary endpoint:</u> Trait Pain Catastrophizing (Trait PC) at 3 months post-treatment. NB: PC is our primary endpoint—and not pain intensity—because PC exerts a negative influence across *multiple* health-related variables within the context of chronic pain. Furthermore, PC is a response to actual and *anticipated* pain, thus underscoring the need to study PC as the primary outcome.

Hypothesis 1a (Superiority): FCR provides durable and substantial reduction in PC compared to HE. Our primary endpoint is PCS score at 3 months, and its within-subject difference from baseline is calculated.

Primary 1a Analyses: The mean difference in the FCR group will be compared against the HE group using 2-sample t-test. We conservatively plan to enroll 231 participants and have 165 completers (55 per group). With this, we achieve 90% power to reject the null hypothesis of equal means when the population mean difference is 5 (63% of that seen in the pain-CBT literature; 45% of that seen in Preliminary Study 1), and 80% power to reject the null when the population mean difference is 4.3 (54% of that seen in the pain-CBT literature; 40% of that seen in Preliminary Study 1), with standard deviation (SD) for both groups of 8, $\alpha = 0.05$, using a 2-sided, 2-sample equal-variance t-test.

Hypothesis 1b (Assay sensitivity / positive control): Pain-CBT provides durable and substantial

reduction in PC compared to HE.

Primary 1a Analyses: Our primary endpoint is PCS score at 3 months, and its within-subject difference from baseline is calculated. The mean difference in the pain-CBT group is compared against the HE group using 2-sample *t*-test. The mean effect of pain-CBT on PCS is about 8 in the literature [21, 71]. As in Hypothesis 1, group sample sizes of 55 each in the pain-CBT and HE groups achieve 90% power to reject the null hypothesis of equal means when the population mean difference is 5 (63% of that seen in literature), and 80% power to reject the null when the population mean difference is 4.3 (53% of that seen in literature), with a SD for both groups of 8 and with a significance level (α) of 0.05 using a 2-sided, 2-sample equal-variance *t*-test.

Hypothesis 1c (Non-inferiority): FCR provides durable and substantial reduction in PC non-inferior to pain-CBT.

Primary 1c Analyses: Group sample sizes of 55 in each group achieve 80% power with margin of non-inferiority 4.3. The true difference between the means is assumed to be 0, the significance level (alpha) of the test is 0.025, and the population standard deviations is 8 in both groups. Given the effect of pain-CBT on PCS is about 8 [21, 71], this non-inferiority margin would imply the single-session FCR retains half of the effect of the 8-session pain-CBT on PCS, a clinically well-accepted level of superiority retention [72-74].

Hypothesis 1d (Global improvement): Reductions in PC provided by FCR is associated with improvements in multidimensional psychometric measures across multiple follow-up time points

Primary 1d Analyses: rmANOVA will be used.

Hypothesis 1e (Improvement in objective measurement of function): Reductions in PC provided by FCR is associated with improvements in objective physical function.

Primary 1e Analyses: This association is measured with correlation coefficient between changes from baseline to primary endpoint in PC and in each of the actigraphy measures for physical activity and for sleep. A sample size of 55 achieves 90% power to detect a correlation coefficient of 0.42, and 80% power to detect a coefficient of 0.37, using a 2-sided hypothesis test with a significance level of 0.05. Multiple comparison adjustments will be made to protect the false discovery rate.

Aim 2: Characterize the mechanistic influence of Daily PC on future pain, function, and Trait PC

We will use multi-level modeling to characterize treatment effects on Daily PC fluctuations, and the impact of Daily PC changes on Trait PC, longitudinal PROMIS measures, and actigraphy (activity and sleep).

Hypothesis 2a (Level-1 PC effect): Daily PC will predict next day changes in pain and activity. Multi-level modeling (MLM) will be used to account for subject-level (level 2) effects in particular the effect of the intervention as well as daily level variations in PC.

Hypothesis 2b (FCR/CBT moderation of Level-1 PC lagged effect): The relationship between daily PC and levels in same day and next day pain and activity is reduced by FCR and CBT interventions compared to the HE group. MLM will be used to test this hypothesis with the intervention as the moderator of daily PC effect on next day pain and activity.

Hypothesis 2c (Level-2 FCR effect): Daily PC mean changes from baseline to 1 month post-treatment will predict mean change in pain, activity, sleep and Trait PC from 1-3 months post-treatment (for the FCR and CBT groups). An analysis of level-2 variance will be performed in the MLM framework.

Hypothesis 2d (Level-1 FCR/CBT skill use effect): Previous day use of FCR / CBT skills will predict next day improvement in Daily PC. MLM will be used to account for subject-level (Level 2) effects in particular daily level variations in use of FCR skills.

9.5.2 Secondary Outcomes

Secondary endpoints: Trait PC at 6 months post-treatment. PROMIS at post-treatment 3 months in each of the core outcome domains for chronic pain clinical trials identified by the Initiative on Methods, Measurement, and Pain Assessment in Clinical Trials (IMMPACT) consensus panel [43] and the NIH Task Force on Research Standards for Chronic Low Back Pain [44] including pain intensity, physical functioning, and emotional functioning. Actigraphy for function and sleep.

9.6 Data Analyses

Specific analyses to be conducted are detailed in Section 9.5 above. We will examine whether there are any between-group differences in the above mentioned variables using a single multivariate GLM. If there are any between-group differences, we will include the variable(s) as covariates in our subsequent analyses.

Subject Adherence and Intention to Treat (ITT) and Per Protocol (PP) Analyses: A portion of subjects will be lost to follow-up. Attrition assumption is typically 25% in the pain-CBT literature [24]. We conservatively estimate a 35% drop-out rate for the pain-CBT group, and 25% attrition for the less burdensome FCR and HE groups. Thus, our study completion target of 55 subjects in each arm indicates a need to (over)enroll a total of 73 subjects for both the FCR and HE groups, and 85 in the pain-CBT group**.** Data analysis will be performed primarily as intention-to-treat (ITT) to protect the effects of randomization from confounding introduced by subject dropout and crossover. Compared to PP analysis, ITT is also considered conservative in the context of superiority hypothesis testing (Hypotheses 1 and 2, which are used to power this study). With regards to missing data, we will attempt to follow all participants, even if they withdraw from allocated treatment. A main analysis will be performed of all valid observed data under a plausible assumption about the missing data. This will be followed by sensitivity analyses that accounts for all randomized patients, to explore the effect of departures from the assumption made in the main analysis. In addition, we note that in the context of non-inferiority hypothesis testing (Hypothesis 1c), the use of ITT instead of being conservative may bias the results toward non-inferiority. We will thus for Hypothesis 1c perform PP analysis as well as ITT analysis. Non-inferiority will be achieved only if both analyses afford the same inference of non-inferiority.

Multi-level Modeling: This will be used to characterize treatment effects on Daily PC fluctuations, and the impact of Daily PCS changes on Trait PC, longitudinal PROMIS measures, actigraphy (activity and sleep).

- Level-1 PC model: We will test the effect of Daily PC on same-day and next-day levels of pain and activity.
- FCR/CBT moderation of Level-1 PC lagged model: We will test the relationships between daily PC and same day and next day pain and activity within groups and between groups.

- Level-2 FCR model: We will test whether daily PC mean changes (baseline to 1 month post-treatment) predict mean change in pain, activity, sleep and Trait PC in the FCR and 8-week CBT groups at months 1-3.
- Level-1 FCR/CBT skills use lagged model: We will test whether previous day and same day use of FCR / CBT skills predict next day improvement in Daily PC.

Actigraphy Analysis Plan: We have substantial experience in analyzing large-scale actigraphy data from the National Health and Nutrition Examination Survey (NHANES) database, where more than 6,000 subjects contributed 7-day per-minute actigraphy data [41]. Our algorithm, discretized multiple adaptive regression (DMAR), is a collection of models that adjusts for population-level differences in physical activities discovered in the CDC database, thus improving the signal to noise ratio for further scientific investigations. This was demonstrated in our work where the adjusted physical activity data demonstrated significant associations between low back pain, physical activity, and obesity [41]. This formed the basis of our development of the Profiles of Physical Performance (PoPP) algorithm that could distinguish subjects with and without pain using actigraphy data [69, 75]. A threshold of 85% completion will be set for inclusion in the analytic dataset.

In accordance with NIH policy, if data from prior studies do not negate strongly the existence of significant differences of clinical or public health importance in the intervention effect between gender and racial/ethnic subgroups a valid analysis of the intervention effect will be performed in these subgroups. If data from prior studies do not strongly support the existence of significant differences in the intervention effect between subgroups, then the analyses need not have high statistical power for detecting clinically meaningful differences.

10. DATA COLLECTION AND QUALITY ASSURANCE

10.1 Data Collection Forms

All questionnaires will be completed by participants in the REDCap Database. If some questionnaires have to be collected on paper due to unforeseen circumstances, the forms will be stored as source data and a member of the study team will enter the data into the REDCap database. A note indicating this occurrence will be made and added to the participant record.
All participants will be identified by a unique study ID number on their corresponding data and case report forms and will not be identified by their name. All CRFs will be stored in REDCap or in binders identified by the Participant ID number and stored in locked cabinets by the study staff Core. All staff will receive training on completing CRFs appropriately, reviewing CRFs for completeness, and maintaining participant confidentiality.

10.2 Data Management

All collected data will be managed by the study staff. As mentioned above, all data will be de-identified and stored in locked cabinets to ensure participant confidentiality.

All data collection forms filled out by the participant will be administered via REDCap. All CRFs will follow the NCCIHtoolbox templates with the Project Number, Participant ID, and Visit Date at the top of the form. The form's heading will be the name of the CRF followed by the name of the study. All CRFs will also have a footer for research personnel completing the form to sign and date it.

10.3 Quality Assurance

10.3.1 Training

All research team members will perform the responsibilities as outlined by the delegation of authority log. Human Subjects Training and HIPAA training will occur as required by Stanford Policy. Additionally, all team members will receive a copy of this document, the Lab Policy Manual, and will be trained directly by the Principal Investigator (PI) on the purpose of the study and their responsibilities. Research staff will be trained on Informed Consent, Phone Screening, and Case Report Form Completion by the study coordinator, who will monitor appropriate conduct and form completion on an ongoing basis, in collaboration with the Division Research Manager and the PIs. Research team meetings will occur frequently with the PI to ensure ongoing understanding by study staff and to address any concerns.

Regulatory and Ethical Training Requirements

The study coordinator will ensure that all study personnel have completed required federal and institutional training, and will maintain documentation of such. The coordinator will assist with the scheduling of training workshops, development of training agendas, and preparation of materials to reinforce ethical research and Good Clinical Practices.

- Human Subjects Protections: Training will include guidelines related to: HIPAA regulations and participant confidentiality, ethical research conduct, and human subjects interactions.
- Conflict of Interest: Compliance with Stanford's conflict of interest policies and ongoing monitoring will also be provided.

10.3.2 Quality Control Committee

The study personnel will conduct internal monitoring of case report form completion and protocol adherence on a quarterly basis. All consent forms will be monitored by the Pain Division Research Manager. Monitoring results will be provided to the DSMB.

10.3.3 Metrics

The metric for the primary outcome measure is self-reported pain catastrophizing. Daily survey data and participant questionnaires will be reviewed by the study staff on a regular basis for completeness.

10.3.4 Protocol Deviations

A major protocol deviation or violation includes any procedure that differs from the IRB approved protocol that was intended to eliminate an immediate hazard to the participant, was harmful, or is possible serious or continue non-compliance by a study staff member.

Major protocol deviations will be communicated by the research staff to the PIs immediately. All events will be communicated to NCCIH within 5 days of the PI learning of the event. A description of the event will be included. The research staff will also submit this information to the IRB.

All minor protocol deviations (those that do not meet the definition of a major deviation and do not affect the interpretation or outcome of the study) will be reported to NCCIH and Stanford IRB annually.

For each participant, a Protocol Deviations Log will be maintained with the participant's record. A comprehensive Protocol Deviations Log will be maintained by the research staff.

10.3.5 Monitoring

Trial Monitoring

Adverse Events, Serious Adverse Events, Unanticipated Problems, and Protocol Deviations will be monitored on a continual basis and reported per information in the relevant sections.

A trained research assistant will monitor participant attendance and adherence, as well as class instructor treatment fidelity.

Additional ongoing monitoring will occur as detailed below. Results will be recorded on the Monitoring CRF. The PI will review and sign-off on the results and any suggested resolutions.

The first 10 participants will have case report forms and consent forms reviewed for accuracy and completion immediately after enrollment and entry into the follow-up period. After the first 10 participants, case report forms and consent forms will be evaluated for completeness every 3 months.

The study team in collaboration with the Pain Division Research Manager will be responsible for monitoring the study and compiling a report of the results, which will be sent to the entire study team and results will be discussed study team meetings. Ongoing issues with data completion will be addressed with the individual team member and re-training may occur as necessary.

Case Report Form Completion

Data discrepancies will be compared to any source data and corrections made by crossing out the original value, providing the correct value, dating, initialing, and providing a reason for the correction (corrections can be made this way in REDCap). Missing data or data discrepancies that cannot be resolved by verifying source data will be left as missing.

Consent Form Completion

Consent forms will be evaluated for completeness of all signatures, required initials, and dates. Any missing signatures will be obtained by mailing the participant a copy of the consent form and requesting the signature with the current date. Any missing study staff signatures will be obtained and dated with the current date. Participants with missing signatures who are unable, or unwilling, to provide missing signature will be withdrawn from the study and data will not be used.

11. PARTICIPANT RIGHTS AND CONFIDENTIALITY

The sponsor should ensure that it is specified in the protocol or other written agreement that the investigator(s)/institution(s) will permit trial-related monitoring, audits, IRB/IEC review, and regulatory inspection(s) by providing direct access to source data/documents.

11.1 Institutional Review Board (IRB) Review

This protocol and the informed consent document and any subsequent modifications will be reviewed and approved by the Stanford IRB and NCCIH.

11.2 Informed Consent Forms

A signed consent form will be obtained from each participant. Participants must be able to understand all the study procedures in order to be successfully consented. Therefore, only English-speakers will be consented for the study. Individuals under the age of 18 will not be included in the study. The consent form will describe the purpose of the study, the procedures to be followed, and the risks and benefits of participation. A signed copy will be given to each participant and this fact will be documented in the participant’s record.

11.3 Participant Confidentiality

Any data, specimens, forms, reports, video recordings, and other records that leave the site will be identified only by a participant identification number (Participant ID, PID) to maintain confidentiality. All records will be kept in a locked file cabinet. All computer entry and networking programs will be done using PIDs only. Information will not be released without written permission of the participant, except as necessary for monitoring by IRB, the FDA, the NCCIH, and the OHRP.

11.4 Study Discontinuation

The study may be discontinued at any time by the IRB, the NCCIH, the OHRP, the FDA, or other government agencies as part of their duties to ensure that research participants are protected.

12. SUPPLEMENTAL PROTOCOL

We propose an additional, secondary studies for this clinical trial. This study will further support the current hypotheses of the study. Please see details below.

Additional treatment arm for the waitlist control group: Upon completion of all study requirements at 6-month post-treatment, participants in the waitlist control group (Health Education: HE) will have an option of participating in a single session FCR class.

12.1 Rationale for the supplemental protocol

For the waitlist control group, HE intervention is expected to offer minimum, if any, benefit in Trait PC reduction. Therefore, participants in the HE will benefit from an active treatment, FCR class. Additionally, this new treatment arm will provide additional data to determine whether FCR class will be superior to HE for Trait PC reduction within the same subjects.

12.2 Objectives:

A) For additional Treatment arm for the waitlist control group, rmMANOVA with multiple time points (the baseline, post-HE, and post-FCR) will be conducted.

- Hypothesis 1a (Superiority within subjects): FCR will be superior to HE for Trait PC reduction.
- Hypothesis 1b (Global improvement): FCR will be superior to HE for longitudinal changes in PROMIS pain intensity and behavior, fatigue, and sleep disturbance.
- Hypothesis 1c (Improvement in objective measurement of function): Reduction in PC provided by FCR is associated with improvements in objective physical function as measured by actigraphy.

12.3 Study Design

Participants who agree to participate in the waitlist control group will undergo the identical FCR and HE treatment protocols and be asked to complete the same online survey at the same time points (baseline and up to six months post-treatment), as indicated in the protocol and schedule of events above.

13. COMMITTEES

In addition to the DSMB, the key personnel of this study will serve as a committee to review and make decisions concerning this study.

14. PUBLICATION OF RESEARCH FINDINGS

Any publication will be made available for review by the key personnel of this study prior to submission.

15. REFERENCES

1. Freburger, J.K., et al., The rising prevalence of chronic low back pain. Arch Intern Med, 2009. 169(3): p. 251-8.
2. CDC, Prevalence and Most Common Causes of Disability Among Adults --- United States, 2005. MMWR, 2009. 58(16): p. 421-416.
3. Wertli, M.M., et al., The Influence of Catastrophizing on Treatment Outcome in Patients With Non-Specific Low Back Pain: A Systematic Review. Spine (Phila Pa 1976), 2013.
4. Severeijns, R., et al., Pain catastrophizing predicts pain intensity, disability, and psychological distress independent of the level of physical impairment. Clin J Pain, 2001. 17(2): p. 165-72.
5. Abbott, A.D., R. Tyni-Lenne, and R. Hedlund, Leg pain and psychological variables predict outcome 2-3 years after lumbar fusion surgery. Eur Spine J, 2011. 20(10): p. 1626-34.
6. Spinhoven, P., et al., Catastrophizing and internal pain control as mediators of outcome in the multidisciplinary treatment of chronic low back pain. Eur J Pain, 2004. 8(3): p. 211-9.
7. Darnall, B.D., Sturgeon, J.A., Kao, M.C., Hah, J.M., Mackey, S.C., From Catastrophizing to Recovery: A pilot study of a single-session treatment for pain catastrophizing. J Pain Res, 2014. 14(7): p. 219-226.
8. Sturgeon, J.A. and A.J. Zautra, State and trait pain catastrophizing and emotional health in rheumatoid arthritis. Ann Behav Med, 2013. 45(1): p. 69-77.
9. Kao, M., Weber, S., Cook, K., Olson, G., Pacht, T., Darnall, B., Weber, S., Mackey, S., Stanford-NIH Pain Registry: Open source platform for large-scale longitudinal assessment and tracking of modern patient-reported outcomes. Journal of Pain, 2014. 15(4): p. S40.
10. Kao, M.C.J., Cook, K., Olson, G., Pacht, T., Darnall, B.D., Weber, S.C., Mackey, S.C., SNAPL-CAT: Catalyzing the rate-limiting step of big data psychometrics with item-response theory and advanced computerized adaptive testing (poster presentation), in American Medical Informatics Associations (AMIA) 2014 Joint Summits on Translational Science. 2014: San Francisco, CA.
11. Commitee on Advancing Pain Research and Care, Relieving Pain in America: A Blueprint for Transforming Prevention, Care, Education, and Research. 2011, Institute of Medicine.
12. Picavet, H.S., J.W. Vlaeyen, and J.S. Schouten, Pain catastrophizing and kinesiophobia: predictors of chronic low back pain. Am J Epidemiol, 2002. 156(11): p. 1028-34.
13. Burton, A.K., et al., Psychosocial predictors of outcome in acute and subchronic low back trouble. Spine, 1995. 20(6): p. 722-8.
14. Sullivan, M.J., Bishop S.R., Pivik, J., The pain catastrophizing scale: Development and validation. Psychol Assess, 1995. 7: p. 524-32.
15. Smeets, R.J.E.M., et al., Physical capacity tasks in chronic low back pain: what is the contributing role of cardiovascular capacity, pain and psychological factors? Disability & Rehabilitation, 2007. 29(7): p. 577-86.
16. Jensen, M.K., A.B. Thomsen, and J. Hojsted, 10-year follow-up of chronic non-malignant pain patients: opioid use, health related quality of life and health care utilization. Eur J Pain, 2006. 10(5): p. 423-33.
17. Papaioannou, M., et al., The role of catastrophizing in the prediction of postoperative pain. Pain Med, 2009. 10(8): p. 1452-9.
18. Martel, M.O., et al., Catastrophic thinking and increased risk for prescription opioid misuse in patients with chronic pain. Drug Alcohol Depend, 2013. 132(1-2): p. 335-41.
19. Linton, S.J., Do psychological factors increase the risk for back pain in the general

population in both a cross-sectional and prospective analysis? Eur J Pain, 2005. 9(4): p. 355-61.

20. Burns, J.W., et al., Do changes in cognitive factors influence outcome following multidisciplinary treatment for chronic pain? A cross-lagged panel analysis. J Consult Clin Psychol, 2003. 71(1): p. 81-91.

21. Williams, A.C., C. Eccleston, and S. Morley, Psychological therapies for the management of chronic pain (excluding headache) in adults. Cochrane Database Syst Rev, 2012. 11: p. CD007407.

22. Burns, J.W., et al., Cognitive factors influence outcome following multidisciplinary chronic pain treatment: a replication and extension of a cross-lagged panel analysis. Behav Res Ther, 2003. 41(10): p. 1163-82.

23. Turner, J.A., S. Holtzman, and L. Mancl, Mediators, moderators, and predictors of therapeutic change in cognitive-behavioral therapy for chronic pain. Pain, 2007. 127(3): p. 276- 86.

24. Thorn, B.E., et al., A randomized clinical trial of targeted cognitive behavioral treatment to reduce catastrophizing in chronic headache sufferers. J Pain, 2007. 8(12): p. 938-49.

25. Rosenstiel, A.K. and F.J. Keefe, The use of coping strategies in chronic low back pain patients: relationship to patient characteristics and current adjustment. Pain, 1983. 17(1): p. 33- 44.

26. Turner, J.A., L. Mancl, and L.A. Aaron, Brief cognitive-behavioral therapy for temporomandibular disorder pain: effects on daily electronic outcome and process measures. Pain, 2005. 117(3): p. 377-87.

27. Sturgeon, J.A. and A.J. Zautra, Psychological resilience, pain catastrophizing, and positive emotions: perspectives on comprehensive modeling of individual pain adaptation. Curr Pain Headache Rep, 2013. 17(3): p. 317.

28. Kao, M., Cook, K., Olson, G., Pacht, T., Darnall, B., Weber, S., Mackey, S., Stanford-NIH Pain Registry: Catalyzing the rate limited step of psychometrics with modern patient-reported outcomes. Journal of Pain, 2014. 15(4): p. S3.

29. Dworkin, R.H., et al., Interpreting the clinical importance of group differences in chronic pain clinical trials: IMMPACT recommendations. Pain, 2009. 146(3): p. 238-44.

30. Osman, A., et al., Factor structure, reliability, and validity of the Pain Catastrophizing Scale. J Behav Med, 1997. 20(6): p. 589-605.

31. Osman, A., et al., The Pain Catastrophizing Scale: further psychometric evaluation with adult samples. J Behav Med, 2000. 23(4): p. 351-65.

32. Van Damme, S., et al., A confirmatory factor analysis of the Pain Catastrophizing Scale: invariant factor structure across clinical and non-clinical populations. Pain, 2002. 96(3): p. 319- 24.

33. Darnall, B.D., Aickin, M., Zwickey, H., A pilot study of inflammatory responses following a negative imaginal focus: Analysis by gender. Gender Medicine, 2010.

34. Edwards, R.R., C.M. Campbell, and R.B. Fillingim, Catastrophizing and experimental pain sensitivity: only in vivo reports of catastrophic cognitions correlate with pain responses. J Pain, 2005. 6(5): p. 338-9.

35. Sullivan, M.J.L., The pain catastrophizing scale: development and validation. Psychological Assessment, 1995. 7(4): p. 524-32.

36. Younger, J., Noor, N., McCue, R., Mackey, S., Low-dose naltrexone for the treatment of fibromyalgia: Findings of a small, randomized, double-blind, placebo-controlled, counterbalanced, crossover trial assessing daily pain levels. Arthritis & Rheumatism, 2013. 65(2): p. 529-538.

37. Younger, J.W. and S.C. Mackey, Fibromyalgia symptoms are reduced by low-dose naltrexone: a pilot study. Pain Medicine, 2009. 10(4): p. 663-72.

38. Sturgeon, J.A., A.J. Zautra, and A. Arewasikporn, A multilevel structural equation modeling analysis of vulnerabilities and resilience resources influencing affective adaptation to chronic pain. Pain, 2013.
39. Ung, H., et al., Multivariate Classification of Structural MRI Data Detects Chronic Low Back Pain. Cereb Cortex, 2012.
40. Younger, J.W., et al., Prescription opioid analgesics rapidly change the human brain. Pain, 2011. 152(8): p. 1803-10.
41. Kao, M.C., Jarosz, R., Goldin, M., Patel, A., Smuck, M., Determinants of Physical Activity in America: A First Characterization of Physical Activity Profile using National Health and Nutrition Examination Survey (NHANES) Phys Med and Rehabil, 2014: p. PMID:24631950.
42. Burns, J.W., M.A. Day, and B.E. Thorn, Is reduction in pain catastrophizing a therapeutic mechanism specific to cognitive-behavioral therapy for chronic pain? Transl Behav Med, 2012. 2(1): p. 22-9.
43. Turk, D.C., et al., Core outcome domains for chronic pain clinical trials: IMMPACT recommendations. Pain, 2003. 106(3): p. 337-45.
44. Deyo, R.A., et al., Report of the NIH Task Force on research standards for chronic low back pain. J Pain, 2014. 15(6): p. 569-85.
45. Smith, G.T., & McCarthy, D. M., Methodological considerations in the refinement of clinical assessment instruments. Psychol Assess, 1995. 7(3): p. 300.
46. Kinser, P.A. and J.L. Robins, Control group design: enhancing rigor in research of mind-body therapies for depression. Evid Based Complement Alternat Med, 2013. 2013: p. 140467.
47. Stoelb, B., Balderson, B., Turner, J.A., Mind-Body Approaches to Pain Management (MAP) Study: CBT Therapist Manual. 2012, Seattle, WA: Group Health Research Institute. (Developed with funding from NIH/NCCAM R01 AT006226; Dan Cherkin, PhD, Principal investigator).
48. Stoelb, B., Balderson, B., Turner, J.A., Mind-Body Approaches to Pain Management (MAP) Study: CBT Patient Workbook. 2012, Seattle, WA: Group Health Research Institute (Developed with funding from NIH/NCCAM R01 AT006226; Dan Cherkin, PhD, Principal Investigator).
49. Ehde, D.M., Dillworth, T. M., & Turner, J.A. , Cognitive-Behavioral Therapy Manual for the Telephone-Delivered Intervention for Pain Study (Unpublished treatment manual). 2012.
50. Balderson, B., Personal communication. January 8, 2014.
51. Turk, D.C., Winter, F., The Pain Survival Guide: How to reclaim your life. 2005, Washington, D.C.: American Psychological Association.
52. Sheehan, D.V., et al., The Mini-International Neuropsychiatric Interview (M.I.N.I.): the development and validation of a structured diagnostic psychiatric interview for DSM-IV and ICD-10. J Clin Psychiatry, 1998. 59 Suppl 20: p. 22-33;quiz 34-57.
53. Younger, J., et al., Development of the Stanford Expectations of Treatment Scale (SETS): a tool for measuring patient outcome expectancy in clinical trials. Clin Trials, 2012. 9(6): p. 767- 76.
54. Cella, D., et al., The Patient-Reported Outcomes Measurement Information System (PROMIS): progress of an NIH Roadmap cooperative group during its first two years. Med Care, 2007. 45(5 Suppl 1): p. S3-S11.
55. Hung, M., et al., The Psychometric Properties of the PROMIS Physical Function Item Bank in Spine Patients. Spine (Phila Pa 1976), 2013.
56. Lai, J.S., et al., How item banks and their application can influence measurement practice in rehabilitation medicine: a PROMIS fatigue item bank example. Arch Phys Med Rehabil,

2011. 92(10 Suppl): p. S20-7.
57. Revicki, D.A., et al., Development and psychometric analysis of the PROMIS pain behavior item bank. Pain, 2009. 146(1-2): p. 158-69.
58. Revicki, D.A., et al., Exploratory and confirmatory factor analysis of the PROMIS pain quality item bank. Qual Life Res, 2013.
59. Fries, J.F., B. Bruce, and D. Cella, The promise of PROMIS: using item response theory to improve assessment of patient-reported outcomes. Clin Exp Rheumatol, 2005. 23(5 Suppl 39): p. S53-7.
60. Nicholas, M.K., Self-efficacy and chronic pain, in 1989 conference of the British Psychological Society. 1989: St. Andrews.
61. Brister, H., et al., Self-efficacy is associated with pain, functioning, and coping in patients with chronic temporomandibular disorder pain. J Orofac Pain, 2006. 20(2): p. 115-24.
62. Jerant, A., et al., Perceived control moderated the self-efficacy-enhancing effects of a chronic illness self-management intervention. Chronic Illn, 2008. 4(3): p. 173-82.
63. Lorig, K., et al., Development and evaluation of a scale to measure perceived self-efficacy in people with arthritis. Arthritis Rheum, 1989. 32(1): p. 37-44.
64. Marks, R., J.P. Allegrante, and K. Lorig, A review and synthesis of research evidence for self-efficacy-enhancing interventions for reducing chronic disability: implications for health education practice (part I). Health Promot Pract, 2005. 6(1): p. 37-43.
65. Zautra, A., et al., Examinations of chronic pain and affect relationships: applications of a dynamic model of affect. J Consult Clin Psychol, 2001. 69(5): p. 786-95.
66. Long, A.C., T.M. Palermo, and A.M. Manees, Brief report: using actigraphy to compare physical activity levels in adolescents with chronic pain and healthy adolescents. J Pediatr Psychol, 2008. 33(6): p. 660-5.
67. Lunde, L.H., et al., Characteristics of sleep in older persons with chronic pain: a study based on actigraphy and self-reporting. Clin J Pain, 2010. 26(2): p. 132-7.
68. Wilson, A.C. and T.M. Palermo, Physical activity and function in adolescents with chronic pain: a controlled study using actigraphy. J Pain, 2012. 13(2): p. 121-30.
69. Smuck, M., et al., Activity Monitoring with Accelerometry Outperforms Self-Reported and Laboratory Assessments of Function in Patients with Lumbar Spinal Stenosis. PM&R, 2013. 5(9): p. S292-S292.
70. Darnall, B.D., Sturgeon, J.A., Kao, M.C., Mackey, S.C., Control Over Catastrophizing: A pilot study of a single-session psychobehavioral intervention for pain catastrophizing (abstract). J Pain, 2014. 15(4): p. S109.
71. Thorn, B.E., et al., Randomized trial of group cognitive behavioral therapy compared with a pain education control for low-literacy rural people with chronic pain. Pain, 2011. 152(12): p. 2710-20.
72. Jones, B., et al., Trials to assess equivalence: the importance of rigorous methods. BMJ, 1996. 313(7048): p. 36-9.
73. D'Agostino, R.B., Sr., M. Campbell, and J. Greenhouse, Non-inferiority trials: continued advancements in concepts and methodology (special papers for the 25th Anniversary of Statistics in Medicine 25(7)). Stat Med, 2006. 25(7): p. 1097-9.
74. D'Agostino, R.B., Sr., J.M. Massaro, and L.M. Sullivan, Non-inferiority trials: design concepts and issues - the encounters of academic consultants in statistics. Stat Med, 2003. 22(2): p. 169- 86.
75. Smuck, M., et al., Does physical activity influence the relationship between low back pain and obesity? The Spine Journal, 2013.
76. Schulz, K.F., et al., CONSORT 2010 statement: updated guidelines for reporting parallel group randomised trials. PLoS Med, 2010. 7(3): p. e1000251.

77. Diener, E., Emmons, R. A., Larsen, R. J., & Griffin, S. (1985). The Satisfaction with Life Scale. Journal of Personality Assessment, 49, 71-75.
78. Cohen, S., Kamarck, T., & Mermelstein, R., "A Global Measure of Perceived Stress," in *Journal of Health and Social Behavior, 24* (1983), 385-396.
79. Bernstein, D. P., Ahluvalia, T., Pogge, D., Handelsman, L. (1997). Validity of the Childhood Trauma Questionnaire in an adolescent psychiatric population. *Journal of the American Academy of Child & Adolescent Psychiatry, 36 (3),* 340- 348.
80. Watson, D., Clark, L. A., & Tellegen, A. (1988). Development and validation of brief measures of positive and negative affect: The PANAS scales. *Journal of Psychology, 54(6),* 1063-1070.
81. Dworkin RH, Turk DC, Farrar JT, et al. Core outcome measures for chronic pain clinical trials: IMMPACT recommendations. Pain 2005;113:9–19.
82. Tracey TJ, Kokotovic AM: Factor structure of the Working Alliance Inventory. Psychological Assessment: A Journal of Consulting and Clinical Psycholology 1989; 1:207–210
83. Devilly, G. J. and T. D. Borkovec (2000). "Psychometric properties of the credibility/expectancy questionnaire." J Behav Ther Exp Psychiatry 31(2): 73-86.
84. Darnall BD, Sturgeon JA, Cook KC, Taub CJ, Kao MC, Rico T, Mackey SC. Development and Validation of a Daily Pain Catastrophizing Scale (Daily PCS) Measure. J Pain. April 2016; 17(4): S20- 21.

16. SUPPLEMENTS/APPENDICES

Appendix 1 – Schedule of Events

Appendix 2 – Informed Consent Templates

Appendix 3 – CBT Manual

Appendix 4 – CBT Workbook

Appendix 5 – CBT Fidelity Rating Sheets

Appendix 6 – FCR PowerPoint Slides

Appendix 7 – FCR Handouts

Appendix 8 – FCR App

Appendix 9 – FCR Fidelity Rating Sheet

Appendix 10 – HE PowerPoint Slides

Appendix 11 – HE Fidelity Rating Sheet

Appendix 12 – Daily PCS Manuscript

Appendix 13 – DSMP

Appendix 14 – Adverse Event Form

Appendix 15 – Serious Adverse Event Form

Appendix 16 – Concomitant Medications Form

Appendix 17 – Unanticipated Problem Form

Appendix 18 – Baseline NRS Form

Appendix 19 – Consent Process Form

Appendix 20 – Demographics Form

Appendix 21 – Inclusion Exclusion Form

Appendix 22 – Medical History Form

Appendix 23 – Participant Tracking/ Randomization and Enrollment Form

Appendix 24 – Screening Visit Checklist

Appendix 25 – Study Completion Form

Appendix 26 - Participant Compensation Scheme

Appendix 27 - Anuradha Roy's CV

Appendix 28 – Valerie Jackson's CV

Appendix 29 – Sue Gritzner's CV

Appendix 30 – Maisa Ziadni's CV

Appendix 31 – Kristen Slater's CV

Appendix 32 – Dennis Keane's CV

Appendix 33 – Shannon Fish's CV

Appendix 34 – Lu Tian's Biosketch

Appendix 35 - Dokyoung (Sophia) You's CV

Appendix 36 – Brief Physical Assessment, Radicular Symptoms
