# Supplementary material for: Comparison of a Single-Session Pain Management Skills Intervention With a Single-Session Health Education Intervention and 8 Sessions of Cognitive Behavioral Therapy in Adults With Chronic Low Back Pain: A Randomized Clinical Trial
Source: JAMA Netw Open. 2021 Aug 16;4(8):e2113401. doi: 10.1001/jamanetworkopen.2021.13401 (PMC8369357; doi:10.1001/jamanetworkopen.2021.13401)
Supplement: Supplement 2. — eTable 1. Between-Group Differences for Posttreatment Pain Catastrophizing Scale Scores (Per Protocol) eTable 2. Minimally Important Difference (MID) for Secondary Outcomes eTable 3. Secondary Outcomes at Baseline and Posttreatment Months 1-3 by Treatment Group With Between-Group Comparisons (Intention to Treat; Adjusted for Additional Baseline Covariates) eReferences [file jamanetwopen-e2113401-s002.pdf]

Supplementary Online Content

Darnall BD, Roy A, Chen AL, et al. Comparison of a single-session pain management skills intervention with a single-session health education intervention and 8 sessions of cognitive behavioral therapy in adults with chronic low back pain: a randomized clinical trial. *JAMA Netw Open*. 2021;4(8):e2113401. doi:10.1001/jamanetworkopen.2021.13401

eTable 1. Between-Group Differences for Posttreatment Pain Catastrophizing Scale Scores (Per Protocol)
eTable 2. Minimally Important Difference (MID) for Secondary Outcomes
eTable 3. Secondary Outcomes at Baseline and Posttreatment Months 1-3 by Treatment Group With Between-Group Comparisons (Intention to Treat; Adjusted for Additional Baseline Covariates)
eReferences

This supplementary material has been provided by the authors to give readers additional information about their work.

© 2021 Darnall BD et al. *JAMA Network Open*.

eTable 1. Between-Group Differences for Posttreatment Pain Catastrophizing Scale Scores (Per Protocol)*

	Between-Group Differences														
	**CBT vs HE**					**ER vs HE**					**ER vs CBT**				
**Post-Treatment Time-point**	Estimate	SE	*p*-value ‡	95% CI		Estimate	SE	p-value ‡	95% CI		Estimate	SE	p-value ‡	One-Sided 97.5% CI	
Month 1	-7.50	1.46	**<0.0001**	10.36	-4.64	-3.88	1.38	**0.0053**	-6.60	-1.16	3.62	1.40	**0.0102**	-∞	6.38
Month 2	-6.74	1.46	**<0.0001**	-9.61	-3.88	-5.05	1.39	**0.0003**	-7.79	-2.31	1.69	1.41	0.2285	-∞	4.46
Month 3	-7.63	1.45	**<0.0001**	10.48	-4.79	-6.09	1.38	**<0.0001**	-8.81	-3.37	1.54	1.40	0.2705	-∞	4.29

Abbreviations: ER, Empowered Relief; CBT, Cognitive Behavioral Therapy; HE, Health Education; SE: standard error; CI = confidence interval
*MMRM analysis was performed with the longitudinal changes in Pain Catastrophizing Scale score from the pre-treatment level as outcomes and adjusted for pre-treatment Pain Catastrophizing Scale score.
‡ Wald P-value. P < 0.05 for bolded pairwise comparisons.

© 2021 Darnall BD et al. *JAMA Network Open*.

eTable 2. Minimally Important Difference (MID) for Secondary Outcomes

Variable	Selected MID	Literature citation to justify MID selection
Pain Intensity	1.5	MID for low back pain is 1.5 to 3.2.[1]
Pain Interference - PROMIS	4.0	Three back pain studies show MID is 3.5 to 5.5[2]
Sleep Disturbance - PROMIS	1.5	MID is 1.0 to 2.0[3]
Physical Function - PROMIS	2.0	MID is 2.0[4]
Depression - PROMIS	3.0	MID is 2.0 to 3.0;[5] others report 3.0 to 3.1[6]
Anxiety - PROMIS	3.0	MID is 2.3 to 3.4[6]
Fatigue - PROMIS	4.0	MID is 3.0 to 5.0[7]
Pain Behavior - PROMIS	5.0	MID is 5.0 (half standard deviation)[8]
Pain Self-Efficacy Questionnaire	5.5	MID for back pain is 5.5[9]
Pain Bothersomeness	1.5	MID not established

© 2021 Darnall BD et al. *JAMA Network Open*.

eTable 3. Secondary Outcomes at Baseline and Posttreatment Months 1-3 by Treatment Group With Between-Group Comparisons (Intention to Treat; Adjusted for Additional Baseline Covariates)*

Outcome Measure (range)<br>Post-Treatment Time-point	CBT vs HE					ER vs HE					ER vs CBT				
	Estimate	SE	*p*-value ‡	95% CI		Estimate	SE	*p*-value ‡	95% CI		Estimate	SE	*p*-value ‡	One-Sided 97.5% CI	
**Pain Intensity (0-10)**<br>**Non-inferiority margin: 1.5**															
Month 1	-0.87	0.31	**0.005**	-1.48	-0.26	-0.54	0.30	0.073	-1.13	0.05	0.33	0.30	0.271	-∞	0.92
Month 2	-0.78	0.31	**0.011**	-1.39	-0.18	-0.87	0.30	**0.004**	-1.46	-0.28	-0.09	0.30	0.769	-∞	0.51
Month 3	-0.94	0.31	**0.002**	-1.54	-0.34	-0.59	0.30	**0.050**	-1.18	0.00	0.35	0.30	0.244	-∞	0.94
**Pain Interference (PROMIS SF 8a) (20-80)**<br>**Non-inferiority margin: 4.0**															
Month 1	-2.83	1.08	**0.009**	-4.95	-0.70	-0.72	1.05	0.496	-2.78	1.35	2.11	1.05	**0.046**	-∞	4.18
Month 2	-3.65	1.08	**0.001**	-5.76	-1.53	-1.76	1.05	0.096	-3.83	0.31	1.89	1.06	0.076	-∞	3.97
Month 3	-4.45	1.07	**<.0001**	-6.55	-2.34	-1.82	1.05	0.083	-3.89	0.24	2.62	1.05	**0.013**	-∞	4.69
**PROMIS Sleep Disturbance (20-80)**<br>**Non-inferiority margin: 1.5**															
Month 1	-0.14	1.36	0.919	-2.81	2.53	-1.86	1.31	0.158	-4.44	0.72	-1.72	1.31	0.189	-∞	0.85
Month 2	-1.50	1.35	0.267	-4.16	1.15	-4.25	1.32	**0.001**	-6.84	-1.66	-2.75	1.32	**0.037**	-∞	-0.16
Month 3	-3.55	1.34	**0.009**	-6.19	-0.91	-5.23	1.31	**<.0001**	-7.80	-2.65	-1.67	1.31	0.201	-∞	0.89
**Pain Self-Efficacy (PSEQ) (0-60)**<br>**Non-inferiority margin: 5.5**															
Month 1	5.31	1.62	**0.001**	2.12	8.50	2.87	1.57	0.067	-0.21	5.95	-2.44	1.57	0.122	-5.42	+∞
Month 2	4.19	1.62	**0.010**	1.01	7.37	2.32	1.58	0.142	-0.78	5.42	-1.87	1.58	0.237	-4.98	+∞
Month 3	6.15	1.61	**0.0002**	2.99	9.32	3.42	1.57	**0.030**	0.34	6.50	-2.73	1.57	0.083	-5.82	+∞

© 2021 Darnall BD et al. *JAMA Network Open*.

**Back Pain Bothersomeness (0-10)**<br>**Non-inferiority margin: 1.5**															
Month 1	-1.11	0.40	**0.006**	-1.89	-0.33	-0.86	0.39	**0.029**	-1.63	-0.09	0.25	0.39	0.521	-∞	1.03
Month 2	-0.84	0.40	**0.035**	-1.62	-0.06	-0.75	0.39	0.056	-1.52	0.02	0.09	0.40	0.817	-∞	0.87
Month 3	-1.31	0.39	**0.001**	-2.08	-0.53	-0.81	0.39	**0.038**	-1.57	-0.05	0.50	0.39	0.203	-∞	1.27
**Pain Behavior (PROMIS SF 7a) (0-10)**<br>**Non-inferiority margin: 5.0**															
Month 1	-1.95	0.86	**0.024**	-3.63	-0.26	-1.83	0.83	**0.028**	-3.45	-0.20	0.12	0.82	0.882	-∞	1.73
Month 2	-1.03	0.85	0.226	-2.71	0.64	-2.48	0.83	**0.003**	-4.11	-0.85	-1.45	0.83	0.081	-∞	0.18
Month 3	-1.93	0.85	**0.023**	-3.60	-0.27	-2.48	0.83	**0.003**	-4.11	-0.86	-0.55	0.82	0.505	-∞	1.07
**PROMIS Fatigue (20-80)**<br>**Non-inferiority margin: 4.0**															
Month 1	-2.05	1.41	0.146	-4.81	0.72	-2.14	1.35	0.114	-4.80	0.51	-0.10	1.35	0.944	-∞	2.56
Month 2	-2.93	1.40	**0.037**	-5.67	-0.18	-2.55	1.35	0.060	-5.21	0.11	0.37	1.36	0.784	-∞	3.05
Month 3	-3.66	1.39	**0.009**	-6.40	-0.93	-1.48	1.35	0.273	-4.13	1.17	2.18	1.36	0.109	-∞	4.84
**PROMIS Depression (20-80)**<br>**Non-inferiority margin: 3.0**															
Month 1	-1.67	1.18	0.159	-4.00	0.66	-1.38	1.15	0.230	-3.63	0.87	0.29	1.14	0.799	-∞	2.54
Month 2	-1.35	1.17	0.250	-3.66	0.95	-1.89	1.14	0.098	-4.14	0.35	-0.54	1.14	0.636	-∞	1.71
Month 3	-2.40	1.17	**0.040**	-4.69	-0.11	-1.94	1.14	0.088	-4.18	0.29	0.45	1.13	0.691	-∞	2.68
**PROMIS Anxiety (20-80)**<br>**Non-inferiority margin: 3.0**															
Month 1	-0.93	1.16	0.422	-3.21	1.35	-0.69	1.12	0.539	-2.89	1.51	0.25	1.12	0.826	-∞	2.45
Month 2	-0.24	1.15	0.837	-2.49	2.02	-1.29	1.12	0.250	-3.48	0.91	-1.05	1.12	0.349	-∞	1.15
Month 3	-3.02	1.14	**0.009**	-5.26	-0.77	-2.47	1.11	**0.027**	-4.65	-0.28	0.55	1.11	0.620	-∞	2.73

© 2021 Darnall BD et al. *JAMA Network Open*.

**PROMIS Physical Function (20-80)** **Non-inferiority margin: 2.0**															
Month 1	0.95	0.90	0.296	-0.83	2.72	0.81	0.87	0.354	-0.91	2.53	-0.14	0.88	0.877	-1.86	+∞
Month 2	1.34	0.90	0.136	-0.42	3.10	0.56	0.87	0.524	-1.16	2.27	-0.78	0.88	0.371	-2.50	+∞
Month 3	2.89	0.89	**0.001**	1.13	4.64	1.64	0.87	0.059	-0.06	3.35	-1.24	0.87	0.154	-2.95	+∞

Abbreviations: ER, Empowered Relief; CBT, Cognitive Behavioral Therapy; HE, Health Education; SE, standard error; CI = confidence interval

* MMRM analysis was performed with the longitudinal changes from the pre-treatment level as outcomes and adjusted for age, gender, race, body mass index, duration of back pain, education, mental health diagnosis, number of co-morbid pain conditions, and baseline values for all outcome variables.

‡ Wald P-value. $P < 0.05$ for bolded pairwise comparisons.

© 2021 Darnall BD et al. *JAMA Network Open*.

eReferences

1. Kovacs FM, Abraira V, Royuela A, et al. Minimal clinically important change for pain intensity and disability in patients with nonspecific low back pain. *Spine (Phila Pa 1976).* 2007;32(25):2915-2920.
2. Amtmann D, Kim J, Chung H, Askew RL, Park R, Cook KF. Minimally important differences for Patient Reported Outcomes Measurement Information System pain interference for individuals with back pain. *Journal of pain research.* 2016;9:251-255.
3. Katz P, Pedro S, Alemao E, et al. Estimates of Responsiveness, Minimally Important Differences, and Patient Acceptable Symptom State in Five Patient-Reported Outcomes Measurement Information System Short Forms in Systemic Lupus Erythematosus. *ACR Open Rheumatol.* 2020;2(1):53-60.
4. Hays RD, Spritzer KL, Fries JF, Krishnan E. Responsiveness and minimally important difference for the patient-reported outcomes measurement information system (PROMIS) 20-item physical functioning short form in a prospective observational study of rheumatoid arthritis. *Ann Rheum Dis.* 2015;74(1):104-107.
5. Kroenke K, Stump TE, Chen CX, et al. Minimally important differences and severity thresholds are estimated for the PROMIS depression scales from three randomized clinical trials. *J Affect Disord.* 2020;266:100-108.
6. Lee AC, Driban JB, Price LL, Harvey WF, Rodday AM, Wang C. Responsiveness and Minimally Important Differences for 4 Patient-Reported Outcomes Measurement Information System Short Forms: Physical Function, Pain Interference, Depression, and Anxiety in Knee Osteoarthritis. *J Pain.* 2017;18(9):1096-1110.
7. Yost KJ, Eton DT, Garcia SF, Cella D. Minimally important differences were estimated for six Patient-Reported Outcomes Measurement Information System-Cancer scales in advanced-stage cancer patients. *J Clin Epidemiol.* 2011;64(5):507-516.
8. Askew RL, Cook KF, Revicki DA, Cella D, Amtmann D. Evidence from diverse clinical populations supported clinical validity of PROMIS pain interference and pain behavior. *J Clin Epidemiol.* 2016;73:103-111.
9. Chiarotto A, Vanti C, Cedraschi C, et al. Responsiveness and Minimal Important Change of the Pain Self-Efficacy Questionnaire and Short Forms in Patients With Chronic Low Back Pain. *J Pain.* 2016;17(6):707-718.

© 2021 Darnall BD et al. *JAMA Network Open*.
