# Supplementary material for: Comparison of a Single-Session Pain Management Skills Intervention With a Single-Session Health Education Intervention and 8 Sessions of Cognitive Behavioral Therapy in Adults With Chronic Low Back Pain: A Randomized Clinical Trial
Source: JAMA Netw Open. 2021 Aug 16;4(8):e2113401. doi: 10.1001/jamanetworkopen.2021.13401 (PMC8369357; doi:10.1001/jamanetworkopen.2021.13401)
Supplement: Supplement 3. — Data Sharing Statement [file jamanetwopen-e2113401-s003.pdf]

Data Sharing Statement

Darnall. Comparison of a Single-Session Pain Management Skills Intervention With a Single-Session Health Education Intervention and 8 Sessions of Cognitive Behavioral Therapy in Adults With Chronic Low Back Pain. *JAMA Netw Open*. Published August 16, 2021. doi:10.1001/jamanetworkopen.2021.13401

Data
Data available: Yes
Data types: Deidentified participant data, Data dictionary
How to access data: Data can be made available upon request and after the specific aims of this NIH-funded trial are completed (expected date 2023)
When available: beginning date: 01-30-2023

Supporting Documents
Document types: None

Additional Information
Who can access the data: (1) data supplied to researchers whose proposed use of the data is approved (2) we can supply the informed consent form upon request
Types of analyses: There are two principal investigators for this NIH-funded trial. The PIs would review requests individually and ensure they do not conflict with existing data requests (and associated analytic plans) or analyses that may be in the pipeline.
Mechanisms of data availability: With a signed data access agreement.
